# A Nitrogen budget for Norway analysis of Nitrogen flows from societal and natural sources (1961–2020)

**DOI:** 10.1371/journal.pone.0313598

**Published:** 2025-02-13

**Authors:** Martin F. Hohmann-Marriott

**Affiliations:** 1 Ruralis, Trondheim, Norway; 2 Centre for Sustainability, University of Otago, Dunedin, Aotearoa, New Zealand; 3 United Scientists CORE, Ōtepoti Dunedin, Dunedin, Aotearoa, New Zealand; National Cheng Kung University, TAIWAN

## Abstract

Nitrogen is a chemical element that is cycled through Earth’s lithosphere, hydrosphere, cryosphere and atmosphere, as well as a crucial component of the molecular machinery of life. Being an intrinsic part of the biosphere, the flow of nitrogen in a country can provide unique insights into the sustainability of a society. This study investigates how nitrogen is used in Norway between 1961 and 2020. Nitrogen inputs from atmospheric deposition, biological nitrogen fixation and from weathering, as well as from synthetic fertilizers are addressed. These sources of nitrogen are used by plants that form the basis of agriculture and forestry. Nitrogen, in the form of protein, is imported to sustain the Norwegian population and the production of animals in agriculture and fisheries. Agricultural livestock is used almost exclusively for domestic consumption, while fish captured and raised in aquaculture are the main sources of exported food. Even though petroleum and wood contain only a small proportion of nitrogen, due to the traded volume, a large amount of nitrogen contained in these goods is exported. Nitrogen is lost through sewage and manure leaching, as well as atmospheric emissions. These emissions are mostly in the form of nitrogen oxides and ammonia, which are released by burning fossil fuels and metabolizing animals, respectively.

## Introduction

Before the advent of life on Earth, most reactive nitrogen (N) was released from volcanoes or produced by lightening in the atmosphere. When life emerged, it is thought to soon have depleted these source of N. In response, life develop a suite of enzymes that convert the inert N in the atmosphere, as well as the N contained in inorganic salts, into reactive, bio-available forms of N. Input from inorganic N through weathering of rock, was greatly accelerated when life gained a foothold on land. Indeed, it has been suggested that the release of N effected by terrestrial bacteria, algae and lichens greatly stimulated carbon dioxide (CO_2_) capture by photosynthesis of aquatic photosynthetic organisms. The consequent loss of the heat-trapping CO_2_ may have been instrumental in establishing the frozen “snowball” Earths that existed between 720 and 635 million years ago [1].

For more than than 10,000 years, humans have manipulated the environment to grow plants for food and to feed animals [2]. Over these long time period, an intimate understanding has evolved, how controlled burning, flooding, plant rotation, and application of manure prevents the depletion of soils.

*“A small proportion of what I take from the soil is replaced by nature (the atmosphere and the rain), the remainder I must restore to the ground*”.

*von Liebig, 1863* [3]

Justus von Liebig contributes the fundamental insight quoted above to Japanese peasants, whom he admires for their agricultural insights and practices. Using state-of-the-art analytical methods, the “father of the fertilizer industry”provided robust insights into how individual chemical elements determine plant productivity. From these studies, N emerged as a main determinant for plant productivity. The increased demand for N was first satisfied by exploiting guano and N-containing mineral deposits. So high was the demand for N, that the exploitation of readily accessible N was (at least in part) the motivation to go to war, including the Chincha Islands War (1864–1866, also known as the “Guano War”) and the War of the Pacific (1879–1883, also known as “Saltpeter War”). However, advances in chemical engineering at the beginning of the 20^th^ century, allowed the the production of synthetic N fertilizer from an omnipresent source of N–air, which consists of around 78% (by volume) of N.

To maintain high plant productivity, today synthetic N fertilizer is extensively used to supplement atmospheric deposition, biological N fixation and rock weathering. The produced plant material can be categorized into food, which directly feeds humans, as well as feed, which is used to feed livestock that in turn are consumed by humans.

Given that only a fraction of N consumed is incorporated into the body of an animal, a large amount of N is excreted. In urban settings, the amount of N contained in human excretions is channeled through sewage systems. Sewage sludge is applied to agricultural lands while soluble N is diluted into rivers. In contrast, N contained in animal excretions is left on pastures or directly applied to cropland. Much of the applied N, however, cannot be recovered, as it leaches into rivers, or is volatilized into the atmosphere as ammonia.

The main form of human-generated atmospheric N emission is NOx, which is released by burning of fossil fuels. Along with ammonia, which is emitted by livestock, NOx is one of the main sources of atmospheric N deposition. Consequently, atmospheric N deposition has increased with industrialization and agricultural livestock production.

A coherent and timeless source of information regarding the flow of N and its role in agriculture and nutrition can be found in the works by Vaclav Smil, including the N cycle [4,5], the role of N in food production [6], and the role of N in nutrition [7,8]. Furthermore, this author has also written a book chapter on the historical perspective of the use of N in Europe [9]. The work by Gu et al. [10] provides useful illustrations that categorize and follow N flows over time on a global scale.

### National Nitrogen budget

The function of a national N budget is to account for the flow of N through a country. The United Nations published a guidance document that details the N “pools”, which should be included as: Energy and Fuels, Material and Products in Industry, Humans and settlements, Agriculture, Forest and semi-natural vegetation, including soils, Waste, Atmosphere and Hydrosphere [11]. The preamble of this document mentions (among others) two motivations for creating an N budget that are relevant to this study: (1) “N budgets respond to the needs of policymakers and national experts to coordinate activities assessing N flows that are potentially adverse to the environment”, and (2) “N budgets are an efficient instrument for visualizing the N cascade and its potential impact and thus help to raise awareness.”

### Previous studies

Studies that portrait comprehensive N budgets on a national level exists for Germany [12,13], Switzerland [14], China [15,16], Denmark [17], and the UK [18,19]. A comprehensive national N budget is also developing for Sweden [20]. The state of national N budgets in the Nordic Countries is summarized by Hellsten and coworkers [21]. Eurostat provides gross values for N input, N output, and N balance (per area), as well as a total N balance and N emissions (gravimetric) for Norway from 1990 until 2016 [22].

For Norway, literature that investigating N flows is fragmented over time, area and scientific discipline. The flow of N in the Norwegian food system was detailed and critically discussed by Blekken and Bakken [23]. The N input into Norway by atmospheric deposition have been estimated [24], as well as the amount of N stored in Norwegian forest soil [25]. There is also published work that provide insight into the flow of N within regions of Norway focused different “pools”. This fragmentation can be demonstrated for publication addressing the flow of N in “hydrology pool”. Here, one study connects shifts in atmospheric N deposition, caused by human activity, to changes in input of N into the Skagerrak, while another study determines N input by two different Norwegian catchments [26]. It can be concluded, that individual scientific publications and their aggregates, do not provide a comprehensive and current view of N flow, at a “pool” or national level for Norway, thus providing motivation to address this gap in our knowledge.

### Covered time period

A comprehensive survey of publicly available data sets was performed, with the intention of determining the quality of data and the time periods covered. The data sets from Food and Agriculture Organization of the United Nations (FAO) stood as as the most comprehensive data sets, with several spanning the time period between 1961 and 2020. The Statistisk sentralbyrå—Statistic Norway provides another rich source of Norway-specific data. However, the latter data sets only cover the recent time period. It was therefore decided to analyze data between 1961 and 2020, so changes over time can be assessed. In addition, data of the most current complete dataset, for the year 2018, is displayed in a diagram that illustrates the flow of N through Norway.

### Goals and scope of this study

This study compose a comprehensive picture of N flow in Norway with the goal of making historical developments accessible, as well as working towards a national N budget. National N budgets are often published in sections. The expertise involved, and the amount of detail processed to create a properly constructed N budget for a country is extensive. The presented study aims to construct a correct picture of the flow of N of Norway, that may, however, not completely satisfy the UN definition of a national N budget.

## Material and methods

### Programs, scripts, data formats and data processing

For basic data manipulation LibreOffice Calc version 7.3.6.2 was used and files were saved in.ods and.csv format. Files in.csv format were generated by saving a.ods worksheet (as.csv), using same file name or the name of the.ods worksheet.

For extensive data manipulation Python version 3.10.6 was used to develop scripts that employ NumPy for bulk numerical processing. Scripts make extensive use of two libraries Table.py and Csv.py, which are included along with the scripts (see S1 Table for description of scripts, and S1 Scripts for the source code) in the supplemental online material.

Data between 1961 and presence were analyzed. Gaps in the data were filled by linear interpolation using existing data points. If data for the most recent 1–6 years was missing the data point from the last existing years were used as an approximation.

Maps were converted between different map projections using G.Projector version 3.0.6 (https://www.giss.nasa.gov/tools/gprojector/), which was also used to obtain with country outlines.

GNU Image Manipulation Program version 2.10.30 was used for image processing, and images were saved in.xfc or.png format. Graphs were constructed using Veusz (version 3.5.3). The manuscript was written using LibreOffice Writer (version 7.3.6.2). Inkscape (version 1.1.2 - 0a00cf5339, 2022-02-04) was used to create Fig 13.

### Geographical parameters

The area of Norway, which is the subject of this study, is Fastlands-Norge (323 808 km^2^), which excludes the islands of Svalbard and Bjørnøya (61 022 km^2^), Jan-Mayen: (377 km^2^) from the total land area of Norway (385 207 km^2^) [27]. Throughout this study we will refer to Fastland Norge as Norway. The coastal waters of Norway are not included in area calculations.

### Land use

To assess the area of land used for agriculture and forestry, two data sets were consulted, the FAO land coverage data set [28] (Table 1), and a recent land cover survey [29]. (Table 2). The FAO data details cropped area as 6068 km^2^ or 2%, tree-covered areas as 133663 km^2^ or 44.55%, and unproductive (artificial surfaces, terrestrial barren land, permanent snow and glaciers) as 27726 km^2^ or 8.6% of Norway.

**Table 1:** Land coverage from FAO [28].

Item	Land area [km^2^]	Land area [%]
Artificial surfaces	1317	0.4
Herbaceous crops	6067	2.0
Grassland	152136	50.7
Tree-covered areas	133663	44.6
Shrub-covered areas	8399	2.8
Shrubs and/or herbaceous vegetation, aquatic or regularly flooded	579	0.2
Terrestrial barren land	11725	3.9
Permanent snow and glaciers	12644	4.2
Inland water bodies	13451	4.5

**Table 2:** Land coverage by Bryn et al. [29].

Item	Land area [km^2^]	Land area [%]
Snow-bed vegetation	19521	6
Alpine heath communities	76723	23.7
Alpine meadow communities	8343	2.6
Boreal deciduous forest	44724	13.8
Broad-leafed deciduous forest	1461	0.4
Pine forest	30470	9.4
Spruce forest	33072	10.2
Peatland forest	12878	4
Wetlands	28777	8.9
Non-forested dry land below the treeline	7088	2
Farm land	12239	3.8
Non-productive areas	30684	9.5
Freshwater	17789	5.5
Total area including freshwater	323771	100

Map data by Aune-Lundberg & Strand et. al [30] showing agricultural areas and forest was used to generate green and red color channel maps. Combining these color channel maps (using the GNU Image Manipulation Program) indicates their co-distribution (appearing as yellow).

### Biome-dependent Nitrogen inputs

Three sources for biome-dependent N inputs can be distinguished: (1) atmospheric N deposition, (2) biological N fixation, and (3) N input from rock weathering. An often used reference for the distribution of biomes is based on a study by the World Wildlife Fund (WWF) [31], referenced here as “WWF biomes”. The area and percentage covered by WWF biomes for Norway is presented in Table 3.

**Table 3:** Area of Norway covered by WWF biomes.

WWF Biome	Area [km2]	Area %
Tundra	199687	61.6
Boreal forests or taiga	102585	31.7
Temperate forests ^a^	21536	6.7

^a^ broad-leaf, mixed forests, and conifer forests.

#### Atmospheric Nitrogen deposition

FAO data estimating the atmospheric N deposition for Norwegian crop land indicates values between from 4.7 (1961) to 7.2 (2020) of kg N ha^-1^ year^-1^ [32]. However, these values—when applied to the total area of Norway (323 808 km^2^), overestimate the the total atmospheric N deposited (181–277 Gg N year^-1^), as the cropped area is located mostly in the Southeast (Østlandet) and temperate costal areas of Norway, which have higher atmospheric N deposition rates than non-coastal and more Northern areas [24]. Therefore, alternative sources for assessing the N input for Norway were consulted. Based on field observation, Hole & Engardt [24] estimated the atmospheric deposition rate for Norway to be 145.2 Gg N year^-1^ (between 1961 and 1990) and 154.1 Gg N year^-1^ (between 1991 and 2021), which is equivalent to 4.48 kg N ha^-1^ year^-1^ and 4.76 N ha^-1^ year^-1^, respectively.

Nitrogen deposition have been measured in Norway in the framework of the Norwegian national monitoring program. Based on these measurements, the total amount of N deposition for Norway have been modeled and the results of this models is reported along with the EMEP chemical transport model (Blake et al. 2023). Here we calculated the average obtained by these models for the time period between 2017–2021, to obtain an N deposition rate of 80.85 Gg N year^-1^.

An alternative approach for estimating atmospheric N deposition is based on the observation that biomes possess specific N deposition rates. The aggregate yearly deposition for Norway can thus be calculated by applying the biome-specific deposition rates to the area of the respective biomes in Norway, and summing up the rates for each biome. The areas of the respective biomes have been obtained using the WWF biome map for Norway.

A first source for biome-specific N deposition is a study by Kwon et al. [33]. After mapping the biomes of this study to equivalent WWF biomes (Ta 4), the total atmospheric N deposition rate for Norway is 74.7 Gg N year^-1^.

A second source for biome-specific N deposition is a study by Houlton et al. [34].

After mapping the biomes of this study to equivalent WWF biomes (Table 4), the total atmospheric N deposition rate for Norway is 33.5 Gg N year^-1^.

**Table 4:** Biome-specific atmospheric Nitrogen deposition rates mapped to WWF biomes.

WWF biomes	Preindustrial [34] [kg N ha^-1^ year^-1^]	Industrial [34] [kg N ha^-1^ year^-1^]	Current [33] [kg N ha^-1^ year^-1^]
Tundra	0.08	0.31	1.14
Boreal	0.35	1.47	2.84
Temperate	1.88	5.7	10.57

#### Biological Nitrogen fixation

The FAO dataset [32] under-reports biological N fixation for agricultural land with biological N fixation between 0 and 0.6 kg N ha−1 year−1 between 1961–2020. Therefore, alternative sources for determining biological N fixation were explored.

A classical source for biome-specific biological N fixation rates is Cleveland et al. [35]. However, more recent estimates of biome-specific biological N fixation [34,36], are substantially lower (Table 5).

**Table 5:** Biome-specific biological nitrogen fixation rates (BNFR) mapped to WWF biomes.

WFF biomes	Preindustrial [34] [kg N ha^-1^ year^-1^]	Industrial[34] [kg N ha^-1^ year^-1^]	Current [36] [kg N ha^-1^ year^-1^]	Recent [35] [kg N ha^-1^ year^-1^]
Tundra	0.46	1.12	3.2	6.1
Boreal	0.71	2.12	2.1	1.8

#### Rock weathering

The world-wide input of N caused by the weathering of rock been estimated, based on the observed rock erosion rates in different biomes [34] (Table 6). The extent of these biomes has been detailed by Dinerstein et al. [37].

**Table 6:** Biome-specific nitrogen input through rock weathering mapped to WWF biomes.

WFF biomes	Preindustrial [34] [kg N ha^-1^ year^-1^]	Industrial [34] [kg N ha^-1^ year^-1^]
Tundra	0.54	1.04
Boreal	0.77	1.47

To calculate the N input of from rock weathering, the area of biomes used for these calculations was reconstructed for Norway.

### Quantification of WWF biomes areas Köppen-Geiger climate zones

For the quantification of biomes, the WWF map with color-coded biomes (geomap format) [31] was used. For the quantification of Köppen-Geiger climate zones, a recent high-resolution (1km x 1km grid) global climate map with color-coded climate zones (geomap format) for the current and future distribution of climate zones [38] was used.

The geomaps were transformed into and equal-area projection (Eckert VI) using the software package G.Projector, which was also used to extract the land area (country outline) of Norway using the same projection. The GNU Image Manipulation Program (GIMP) was used to mask the biome and bio-zone map with the map of the country outline. The select-by-color function in combination with the histogram function in GIMP was used to determine the number of pixels representing each biome/climatic zone. The pixel counts for each climate zone was used to calculate the percentage that this biome/climate zones covered in relation to the pixel count of the country outline mask. The results of following the procedure outlined above are shown for the WWF biomes in Table 7, and for the Köppen-Geiger climate zones in Table 8.

**Table 7:** Area of Norway assigned to WWF biomes.

WWF biomes	Recent (2001) land area [%]
alpine/tundra	61.7
boreal	31.7
temperate	6.7

**Table 8:** Area of Norway covered by Köppen-Geiger climate zones (present and future) in per cent of Norway Fastland.

Köppen-Geiger Climate zone identifier	Present land area [%] (1980–2016)	Future land area [%] (2071–2100)
EF (Polar)	4.3	0
Dsc ET (subartic/tundra)	28.09	1.3
Dfc (boreal)	56.23	41.4
Cfa,Cfb,Cfc (temperate) Dfa, Dfb (hemiboral)	11.41	57.4

Abbreviations: EF: Polar–ice cap, ET: Polar–tundra, Dsc: Continental–dry summer–cold summer, Dfa: Continental–no dry season–hot summer, Dfb: Continental–no dry season–warm summer, Dfc: Continental–no dry season–cold summer, Cfa: Temperate–no dry season–hot summer, Cfb: Temperate—no dry season–warm summer, Cfc: Temperate—no dry season–cold summer.

### Mapping Köppen-Geiger climate zones to WWF biomes

The WWF biomes (S2 Fig) were mapped to Köppen-Geiger climate zones, resulting in the data presented in (Table 9).

**Table 9:** Mapping of Köppen-Geiger climate zones to WWF biomes.

Köppen-Geiger code	Köppen-Geiger description	WWF biomes
Cfb Cfc	temperate	temperate
Dfb	humid continental	temperate
Dsc Dsb	subarctic	boreal
Dfc	boreal	boreal
ET	tundra	alpine / tundra
EF	frost	polar desert

Using the assignment of climate zone to biomes the following biome-equivalent areas were calculated (Table 10). Abbreviations: EF: Polar–ice cap, ET: Polar–tundra, Dsc: Continental–dry summer–cold summer, Dsb: Continental–dry summer–warm summer,

Dfa: Continental–no dry season–hot summer, Dfb: Continental–no dry season–warm summer, Dfc: Continental–no dry season–cold summer, Cfb: Temperate—no dry season–warm summer, Cfc: Temperate—no dry season–cold summer,

**Table 10:** Area of Norway Köppen-Geiger climate zones mapped to WWF biomes for present (1980–2016) future distribution (2071–2100) using mapped biomes (Table 9).

WWF biome	Present land area [%] (1980–2016)	Future land area [%] (2071–2100)
temperate	11.41	57.35
boreal	56.23	41.38
alpine / tundra	28.09	1.27
polar desert	4.27	0.00

### Denitrification

The loss of bound nitrogen through denitrification is here calculated by using reported values for denitrification observed in Western European coniferous forests (~0.57 kg N ha^-1^ year^-1^) [39]. This rate is applied to the soil covered area of Norway (total area– 8.6% unproductive land area)

38 520 700 ha * (1–0.086) * 0.57 * kg N ha^- 1^ year^-1^ = ~20.1 Gg N year^-1^

### Synthetic Nitrogen fertilizer

Data for synthetic N fertilizer production, trade and use in domestic agriculture between

1961 and 2020 are based on FAO dataset [40]. The data was parsed into time series and the unit of the data set (metric tonnes of N), and were converted into grams of N using the script (S1 Script: SynthetheticFertilizer.py).

### Fossil fuel

Quantities for domestically-produced, as well as imported and exported fossil fuels (petroleum, coal), were obtained from the Energy Information Administration data [41]. Petroleum and coal contain N as constituents of organic molecules, which when burned turn mostly into NOx. Additional NOx is produced during combustion of fossil fuels by combining atmospheric N_2_ with atmospheric O_2_.

The produced and traded coal resources (bituminous coal) is a high grade coal, and (metallurgical coke) is also obtained from high quality coal by eliminating contamination that can be out-gassed, including N-containing molecules. For further calculations, the N content of metallurgical coke is there set to 0% while the N content of bituminous coal is set to 2.0% [42] a value that aligns well with older literature [43] citing 1.7%. Thus, when considering production, the N content of metallurgical coke is considered to be 2% (as it is derived from bituminous coal), while for import and export the N content of bituminous coal is 0%.

For petroleum, an N content of 0.4% was determined from the average of crude petroleum samples from the Norwegian Continental Shelf [44]. Quantities of oil reported as “1 barrel per day” were converted into the gravimetric metric units (48.80 tonnes per year) using published conversion metrics [45].

The script Fossil_Fuels.py (S1 Scripts) was used to calculate the amount of N in petroleum and coal.

### Forestry

#### Production, export and import

Volumetric production of wood for industrial use (industrial roundwood) and wood fuel are recorded without gaps between 1961 and 2020 [46] and 1961 and 1996 [47]. These figures represent the entire stock of wood that has been harvested.

Volumetric (round wood, pulp) and gravimetric (other forestry products) quantities for imported and exported wood products were obtained using the FAO dataset [46]. Charcoal was excluded from N content calculations, because N-containing molecules are broken down and the resulting NOx is lost into the atmosphere when charcoal is produced.

#### Nitrogen content of dry wood

The N contents of dry wood for coniferous (Norway spruce, *Picea abies*) was set to 0.14% (w/w) and non-coniferous wood (Red Oak, *Quercus rubra*) was set to be 0.17% in accordance with literature [48]. Wood characteristics in regard to N content and weight were assigned to coniferous wood for export and imported forestry products where the type of wood could not be directly inferred.

#### Fresh and dry weight

The “wood basic density” (dry weight / green m^3^) for Norwegian logs was set to 400 kg/m^3^ (conifer) and 500 kg/m^3^ (non-conifer) [49]. These values in conjunction with the N content of dry wood (see above) can be used to determine the N content of reported logs. The fact that the value of the “Norwegian m^3”^ is 2.5% below the standard “FAO m^3^” value [50] was ignored. The same value was also used for other wood products with the exception of pulp, which was set to 0%. This is due to the treatment of pulp. Consequently paper has also a very low N content [51], and was therefore set to 0% for import/export.

The script Forestry_Products.py (S1 Scripts) was used to calculate the N in forestry products.

### Fisheries

Fisheries data was obtained from FAO fisheries “Capture Production and Global Aquaculture Production” data sets [52,53] spanning the years 1961 to 2020 for “Fisheries” and “Aquaculture”. For analysis, the main items in both datasets were left unchanged, while minor items were aggregated into the category “other”. For capture the category “other” comprises “Aquatic Animals NEI”, “Cephalopods”, “Freshwater and Diadromous Fish”, “Marine Fish NEI”, and “Molluscs excl. Cephalopods”. For aquaculture the category “other” comprises “Aquatic Plants”, “Crustaceans”, “Marine Fish NEI”, and “Mollusks excl. Cephalopods”.

The amount of N contained in fisheries products was calculated assuming a protein content of 75% of caught (fresh) body weight. Furthermore, product-specific N content values were applied to calculate the total N content (contained in S2 Table). On this basis, the N content for fisheries products were calculated using the python scripts (S1 Scripts: Fisheries_Aquaculture.py, Fischeries_Catch.py, Fischeries_Total.py).

### Aquaculture feed

The amount of protein used to feed salmon and rainbow trout, the two species that dominate Norwegian aquaculture was 732127 and 43842 metric tonnes (total 775969 metric tonnes) [54,55]. Assuming an N content of 16% (Jones’ factor, see “Food” section for more detail), the total amount of N used for salmon and trout feed is 124 Gg N.

### Non-food agricultural products

Production and trade of non-food items is disclosed in the FAO dataset [56] with spans the years 1961–2020. This dataset lists abaca, brans, copra cake, cottonseed/cottonseed cake, groundnut cake, oilseed cakes, palm kernels, rape and mustard cake, soya bean cake, sunflower seed cake, being produced and traded. For all these cakes the N content values of cold pressed cakes have been used [57] and are included in S2 Table. The N content was computed for plant seed cakes and fibre products (including plant and animal fibers, such as wool) using scripts (S1 Scripts: NonFood_Domestic.py and NonFood_ImportExport.py).

### Pasture feed and fodder

Pasture feed and fodder for livestock was assessed using two sources of data. Domestic production data for forage and non-forage feed data (2000–2020) was provided by Statistics Norway [58]. Forage items that are part of this data are crops for green fodder and silage, annual rye-grass, green forage mixtures/grain crops, forage rape, marrow-stem kale etc., and hay. The non-forage item “potatoes” is a food item, as the same quantities listed [58] are listed in FAO dataset [59] as food. The N content of these items are contained in the S2 Table, with most of these values sourced from the feedipedia data [60].

For determining the quantities of import and export forage and non-forage feed the FAO data that spans the years (2000–2020) was consulted [61]. Only “hay for forage, and “other forage products, n.e.c.” were traded forage products, and “gluten feed and meal”, “vegetable products for feed n.e.c.” were traded non-forage products. The N content of these items is contained in the S2 Table.

All animal feed items are reported gravimetrically and were classified as forage and non-forage. The N content for the items cited are also presented in the N content table (S2 Table) N in feed was calculated using the scripts Feed_Production.py

Feed_ImportExport.py (S1 Scripts) for production and import/export, respectively.

### Live animal import and exports

Input and export of life animals is reported by FAO data [61]. To estimate the total N content, of imported an exported animals, life weights of animals were estimated [62] (Table 11). This life weight (m_live_) was used as the basis for calculating protein contained in these animals. Applying a factor of 0.7 provides the the “dressed weight” of the animals [63]. The calculated dress weight was multiplied by the factor 0.18 (protein weight /meat weight) and the factor 0.16 (N weight / protein weight; Jones’ factor, see “Food” section for more detail) to obtain the N content of the animal.

m_N_ = m_live_ * 0.7 * 0.18 * 0.16

**Table 11:** Animal weights used for calculating freshweight.

Animal	Animal weight [kg]
ass^1^	125.0
cattle^1^/buffalo^2^	175.0
chicken^1^	2.5
horse^1^	200.0
mule and hinnie^1^	175.0
sheep / goat^1^	25.0
turkey^2^	7.5

^1^ data from FAO [62].

^2^ own estimated.

### Livestock manure

The FOA dataset [64] was used to calculate N flux from manure.

The amount of N that is retained (not lost to the environment is calculated by subtracting the leached and volatile N amounts that were left on the pasture (p) and applied after treatment (a).

N_retained_ = (N_p_−Np-leached−N_p-volatile_) + (N_a_−N_a-leached_−N_a-volatile_)

The total loss of N from manure was calculated by subtracting the amount of N retained from amount of N excreted.

N_loss_ = N_excreted_−N_retained_

The percentage of retained N is the fraction is:

%P_retained_ = (N_excreted_ / N_retained_) * 100

FAO provides dataset information [65] that details the calculation of parameters.

### Cropland nutrient budget

A FAO dataset [32] was used to assess N inputs and losses on cropped land. The cropland nutrient budget data details estimated N inputs (through synthetic fertilizer, application of manure, biological N fixation and atmospheric deposition) as well as N losses (through crop removal, volatilization and leaching). The amount of Manure applied to land and lost was calculated using scripts (S1 Scripts: Manure.py, Manure_Animal.py).

### Food

The FAO data set [59], which is divided in two sections (1961–2013) and (2010–2019) was combined using a script (S1 Scripts: FAOFoodBalance_Combine.py), with 2009/2010 as the border of the two data sets.

#### Protein content

Food category-specific protein content was hand-curated using the USDA food database (FoodDataCentral) [66] or sources indicated in the constructed file (S2 Table) taken into account Norway-specific preferences for the selection of food items. These were cross-checked with FAO tables [67].

The protein content was used to calculate the protein-dependent N content of proteins using Jones’ Factor (mass_N_ = mass_Protein_ / 6.25) [68], although the application of this established conversion factor is not without controversy [69], due to food-specific amino acid composition.

#### N Content—non-protein nitrogen

The N content of food items is higher than the N contained in protein itself due to the presence of nucleic acids, and other N-containing organic molecules [70]. This non-protein N was set to 15% and N content of the food items was calculated accordingly [67].

#### Protein supply, total, and by category

The domestic supply, import and export of N over time was analyzed by splitting the protein-containing items into four categories (1) animal-agriculture, which includes mammals and birds; (2) animal-fisheries, which includes fish, aquatic, invertebrates and mammals (3) dairy and egg products and and (4) plant products. Categories are indicated in the S2 Table along with the N content of every food item. The N content of produced, imported and exported food was calculated using the scripts Food_Domestic.py and Food_ImportExportProductionSupply.py (S1 Scripts).

#### Protein consumption per population and person

The protein supply for the total Norwegian population and the supply on a *per capita* basis was calculated using the protein contents (protein [g] / 100 [g] food) of food items (S1 Scripts: Food_ProteinConsumption.py).

### People

#### Migration

The amount of N contained in people moving in and out of Norway can be assessed [71]. Using the same parameters that were used for life animals (assuming a weight of 75 kg), net migration (18103 people) results in an N input of 27 Mg in 2018.

#### Emission of Nitrogen as Ammonia, through skin and by breathing

A value of 0.8 mg ammonia emission for an adult person^-1^ hour^-1^ has been determined [72]. Using the atomic fraction of N in ammonia (0.833, see section: Nitrogen Oxides and Ammonia) and the number of people in Norway (~5 M), a value for the yearly N missions be humans can be calculated:

0.8 * 10^−3^ g NH_3_ * person^-1^ hour^-1^
* 0.833 g N NH_3_^-1^
* 5 *10^6^ person
* 365 days / year^-1^
* 24 hour day^-1^
= ~29.2 Mg N year

### Sewage and sludge

Data for N loss between 2002 and 2021 for sewage was obtained from the Statistic Norway [73]. The N loss is composed of loss from the sewage stations as well as loss by pipe leakage. Data for N loss between 2015 and 2021 for sewage sludge was obtained from Statistics Norway [74]. A publication that accompanies these datasets for 2018 was used to gain insights into the flow of N in sewage and sewage sludge [75].

### Waste

Data for waste between 2012 and 2021 was available from Statistics Norway [76].

Types of waste that were not considered to contain substantiation amounts N: paper and cardboard (793 kt), glass (144 kt), metals (858 kt), EE waste (129 kt), concrete and bricks (1169 kt), plastics (276 kt), textiles (6 kt), “discarded vehicles (231 kt), radioactive waste (0 kt), slightly polluted soil (2593 kt), other (1427 kt), cinders, dust bottom ash and fly ash (647 kt).

The N-containing waste-type “sludges (236 kt)” was considered equivalent to “dry sewage sludges (111.7 kt)” and the N content is calculated in the *Sewage* section of this study.

The N-containing waste types “wetorganic waste (585 kt)”, “park and garden waste (178 kt)”. “wood waste (769 kt)” and 50% of “Mixed Waste (2762 kt)” [77] and “rubber (64 kt)” were consolidated into “Biowaste” (totaling 2799 kt) and assigned an N content of 9.5g / kg [78] resulting in a total N content 28.3 Gg N.

### Nitrogen oxides and ammonia

Data for nitrogen oxides (NOx) and ammonia (NH_3_) emissions into the air was obtained from the Statistisk sentralbyrå data [79].

NOx is a mixture of components, with NO being the dominant component (95%) while NO_2_ makes the majority of the remaining components [80,81] The mass (m) of N in NOx and ammonia emissions was calculated using the following formulas:

N/NO = m_N_ / (m_N_ + m_O_) = 14/(16+14) = 0.47
N/NO_2_ = m_N_ / (m_N_ + 2 m_O_) = 14/(16+14*2) = 0.30
m_TotalN_ = m_NOx_ * (0.95 N/NO + m_NOx_ * 0.5 N/NO_2_)
N_fraction_ = (0.95 N/NO + 0.5 N/NO2) = 0.44
m_N-NOx_ = m_NOx *_ N_fraction_ = m_NOx_ * 0.44
N/NH3 = m_N_ / (m_N_ + m_O_) = 14/(14+1+1+1)
m_N-NH3_ = m_NH3_ * N/NH3 = m_NH3_ * 0.82

The amount of N contained in and NOx and NH_3_ has been calculated using the script Atmosphere_NOxNH3.py (S1 Scripts).

## Result and discussion

The *Result and Discussion* section is organized into two parts. In the first part, the flow of N in Norway is explored by using comprehensive data sets, most of which spanning the time period from 1961–2020. This time period was chosen as it included complementary data sets from the Food and Agriculture Organization of the United Nations (FAO) and the the Statistisk sentralbyrå—Statistic Norway. Here, each originally reported data set item has been analyzed to obtained the amount of N contained within this item. The flows of N are discussed within each data set. The second part composes N-content data into a concurrent view of N flows within Norway.

The N content and flow rates are displayed in graphs using power of 10 notation, to directly indicate magnitudes, that may change between panels within one graph. For the discussion of data within the text the SI unit prefix G(iga) is used to indicate the magnitude of the units of displayed data items. In order to make it easier to compare different data sets, all data is graphed between 1961 and 2020, even when some data sets do not cover these time periods and consequently, graphs may appear mis-framed.

### Land use

The geographic location of Norway and geological history, make Norway a country where farming is a demanding endeavor. During the last ice age Norway was stripped of much of fertile soil through the relentless grinding of glaciers. Consequently, small fertile areas for agriculture are often located within fjords. A larger area of arable land is available in Østlandet that spans the counties Vestfold og Telemark, Viken, Oslo and Innlandet. Valuable agricultural land is also located along the Atlantic coast, with intensely cultivated areas in Rogaland and Trøndelag. Forrest covers most of Norway with the exception mountainous areas.

Due to the short growing seasons, grain can only be reliably produced, as far North as Trøndelag, even in the mild temperatures afforded by the Atlantic along the coast. The extend of area used for crops, pasture and meadows remains remarkably constant between 1961 and 2020. The area used for growing crops is around 2.1%, while the area allocated for pasture and meadows is around 1.25% of Norway. The total area used for agriculture is therefore around 3.35%, of which 2.55% are considered in active use.

Forest cover is around (31.4% of Norway) (1991–2020), with 99.1 percent of forest in Norway naturally regenerating and about 0.9% planted, and 18.2% considered productive forests.

The area of land considered unproductive (including artificial surfaces, terrestrial barren land, permanent snow and glaciers) is around 9% (see Material and Methods, Tables 1 and 2).

### Biome-dependent Nitrogen inputs and losses

It has been recognized that N input from biological N fixation [35], as well as atmospheric deposition, and rock weathering [34] are biome-dependent.

It is tempting to calculate N inputs from biological N fixation, atmospheric deposition and rock weathering using the specific N-inputs determined from different biomes. The close relationship between biomes and climate zones [82] can even be used to transpose these biome-dependent N inputs using recent high-resolution climate zone data [38]. However, it must be acknowledged that such calculated biome-dependent N-flows carry much uncertainty, as they are averages obtained from experimental data for a large variety of environments. For example, N inputs from rock weathering is certainly dependent on the type of rock present in a certain biome. Furthermore, the quality of soil may be different in Norway from soil in Siberia, even if the soils are located in the same climatic zone. Thus the following estimates for N-inputs should be seen with extreme caution.Using biome-based calculations the total input from rock weathering totals ~178.2 Gg N year^-1^ between 1980–2016. To my knowledge there is no other source for an estimate for biome-based input from rock weathering for Norway that can be compared the rate calculated here.

The biome-based atmospheric N-deposition component is estimated to total ~ 50.7 Gg N year^-1^_,_ which is only only ~1/3 of N-deposition reported by Hole et al. [24], who estimated atmospheric N deposition to be 145.2 Gg N year^-1^ and 159.7 Gg N year^-1^ based on models that use local observations. A more recent study [83] used different model assumption in connection with N deposition data obtained by the Norwegian National Monitoring Program. The total amount of atmospheric N deposition for Norway have been modeled and the results of this models is reported along with calculations based on EMEP models. Here, we calculated the average obtained by these models for the time period between 2017–2021, to obtain an N deposition rate of 80.85 Gg N year^-1^. The basis of this average are models based on observational data (47.2, 116.1 and 79.3 Gg N year^-1^)_._

Biological N fixation is dependent on the growth rate of plants and the bacteria they host. The biome-based estimates for biological N fixation rate is 82.4 gG N year^-1^_._ Unfortunately, I am not aware of any study that also reports the total N input from biological N fixation for Norway.

Chemically-bound nitrogen is lost through denitrification. This complex process is mediated by microbes that convert nitrate into dinitrogen (N_2_) and to a lesser extent to nitrous oxide (N2O) and nitric oxide (NO). As denitrification is dependent on many parameters, including presence of nitrogen components in soil and soil pH, as well as climatic conditions, the total amount of bound nitrogen lost within Norway is very hard to estimate. Furthermore, no definitive methods for measuring N loss through denitrification has been established [39]. A very rough estimate has been obtained by multiplying the soil-covered area of Norway with the average of denitrification observed for Western European coniferous forests soils (20.1 Gg N year^-1^) [39]. This emission has been assigned as an emission from the forestry area to the atmosphere.

### Synthetic Nitrogen fertilizer

Conversion of the inert atmospheric N into biologically reactive N has been the domain of N-fixing bacteria for eons. Humans only gained access to non-biological methods for N fixation recently. A Norwegian contribution is the Birkeland–Eyde process in the early 1900s [84], which imitates the activation of atmospheric N through lightning. While the Birkeland–Eyde process used the abundantly available Norwegian hydropower, the Haber-Bosh [85] process, that replaced it, uses hydrogen derived from hydrocarbons as the source of energy. Being able to produce hydrogen and hydro energy cheaply, Norway remains an important producer of synthetic N fertilizer.

**Fig 1:**
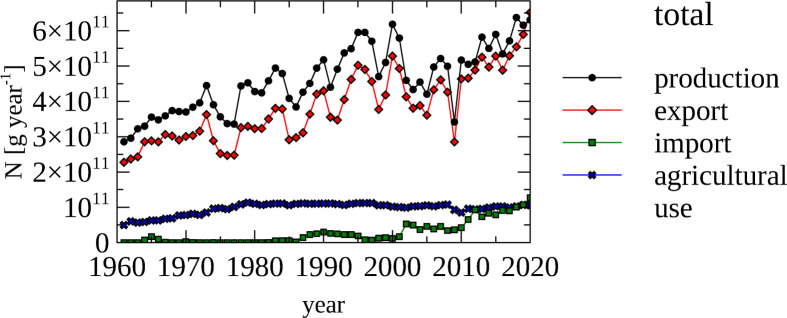
Synthetic nitrogen fertilizer—Nitrogen content of synthetic nitrogen fertilizer (production, export, import and domestic use).

Fig 1 provides a timeline of the amount of synthetic N fertilizer produced, traded and used in domestic agriculture between 1961 and 2020 based on the FAO dataset [40]. The production of synthetic N fertilizers more than doubled from 288 Gg N year^-1^ to 643 Gg N year^-1^ between 1961 and 2020. Most of the produced N is exported (70%-90%), and only in the mid 2000s synthetic N fertilizer is imported in quantities larger than 40 Gg N year^-1^. The agricultural use N fertilizers more than doubled from 50 G N year^-1^ to 120 Gg N year^-1^ between 1961 and 1980, and slightly decreased to the present day use (~110 Gg N year^-1^). The large extent of synthetic N fertilizer use in agriculture becomes clear, when considering that only ~ 178.2 Gg N year^-1^ is the current N input from biome-specific N inputs (as discussed in the preceding section).

The total amount of production, export and import and the amount used in agriculture are displayed between 1961 and 2020.

### Fossil fuels

The discovery of petroleum in the Norwegian shelf transformed the Norwegian economy. The first oil field came on line in 1969 and successive discoveries of other oil fields made Norway the leading oil producer in Norway. In addition to oil, gas was also discovered, thus diversifying the exploitable fossil energy resources.

Fossil fuels are derived from organic matter that has been kept in oxygen-depleted environments. Due to their biological origin, petroleum and coal contain N. Norwegian petroleum contains only around 0.4% (on weight basis) [44], while the N concentration of coal is about 2% (on weight basis) [42]. Natural gas, however, almost exclusively contains inorganic N_2_ (1–0.6%) [86], as organic N is predominately contained in larger organic molecules that have affinity with the co-located coal and oil. Data for the production, export and import of fossil fuels spanning the years 1980 to 2020 [41] was used to calculate produced and traded N from fossil fuels.

**Fig 2:**
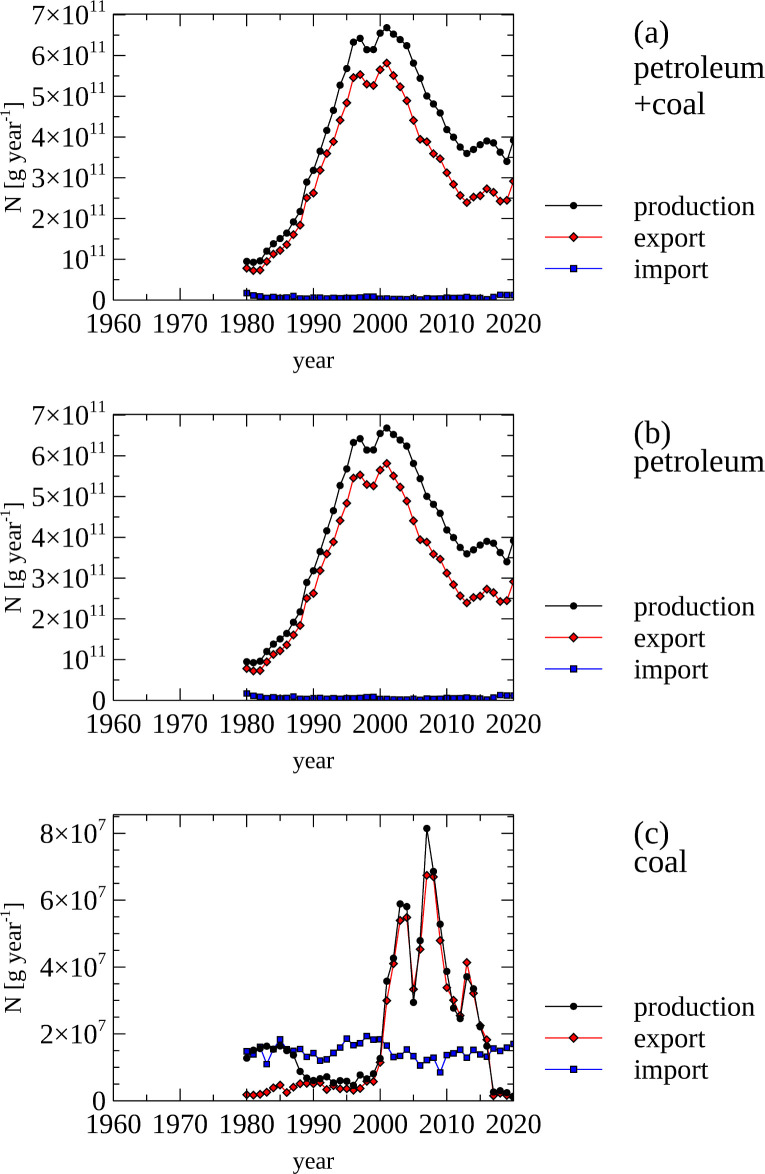
Fossil fuels—Nitrogen content of fossils fuels (produced, imported and exported). (a) Production, export and import of nitrogen in fossil fuels (and coal). (b) Production, export and import of nitrogen contained in petroleum.(c) Production, export and import of nitrogen contained in coal. Note the difference in scale between panels a & b and panel c.

Petroleum is the main source of N in traded fossil fuels (Fig 2, panel a & b) for Norway. The production of petroleum commenced in the late 1970 and reached a maximum between the mid 1990s to mid 2000s, reflected by a peak of petroleum derived N production around 650 Gg N year^-1^ during this time period. More than 75% of petroleum is exported. The domestic consumption of fossil fuels and the concomitant release of N in the form of NOx is captured as emissions (see section: Nitrogen Oxide and Ammonia Emissions).

Coal only contributes a small component of N within fossil fuels in Norway (Fig 2, panel c). There is a steady import of coal of between 18 Mg year^-1^ and 20 Mg N year^-1^ between 1975 and 2020. During 2020 and 2015 coal with an N content averaging 50 Mg N year^-1^ were produced and mostly exported.

### Forestry

*“Agriculture differs essentially from the cultivation of forests, inasmuch as its principal object consists in the production of nitrogen under any form capable of assimilation; whilst the object of forest culture is confined to the production of carbon*”

*von Liebig,1840* [87]

Wood contains a small amount of N that varies with the type of wood. For conifers the N content on a weight to weight basis is 0.14%, while trees featuring broad-leaves have higher N content (0.17%) [48].

The Norwegian forest industry harvests large quantities of trees, and thus a sizable amount of N is harvested, despite the low N content of wood. The harvested wood is classified as industrial logs, which are the basis for related industries (e.g saw mills, paper), and fuel wood used for heating.

Two datasets were used to determine the total amount of wood harvested for every year between 1961 and 2020 [46] and 1961 and [47]. The data show very similar amounts in the overlapping time periods (S3 Fig). The total volumetric amount of trees felled is between 75 Mm^2^ year^-1^ (1960s) and 120 Mm^2^ year^- 1^ (2020s).

**Fig 3:**
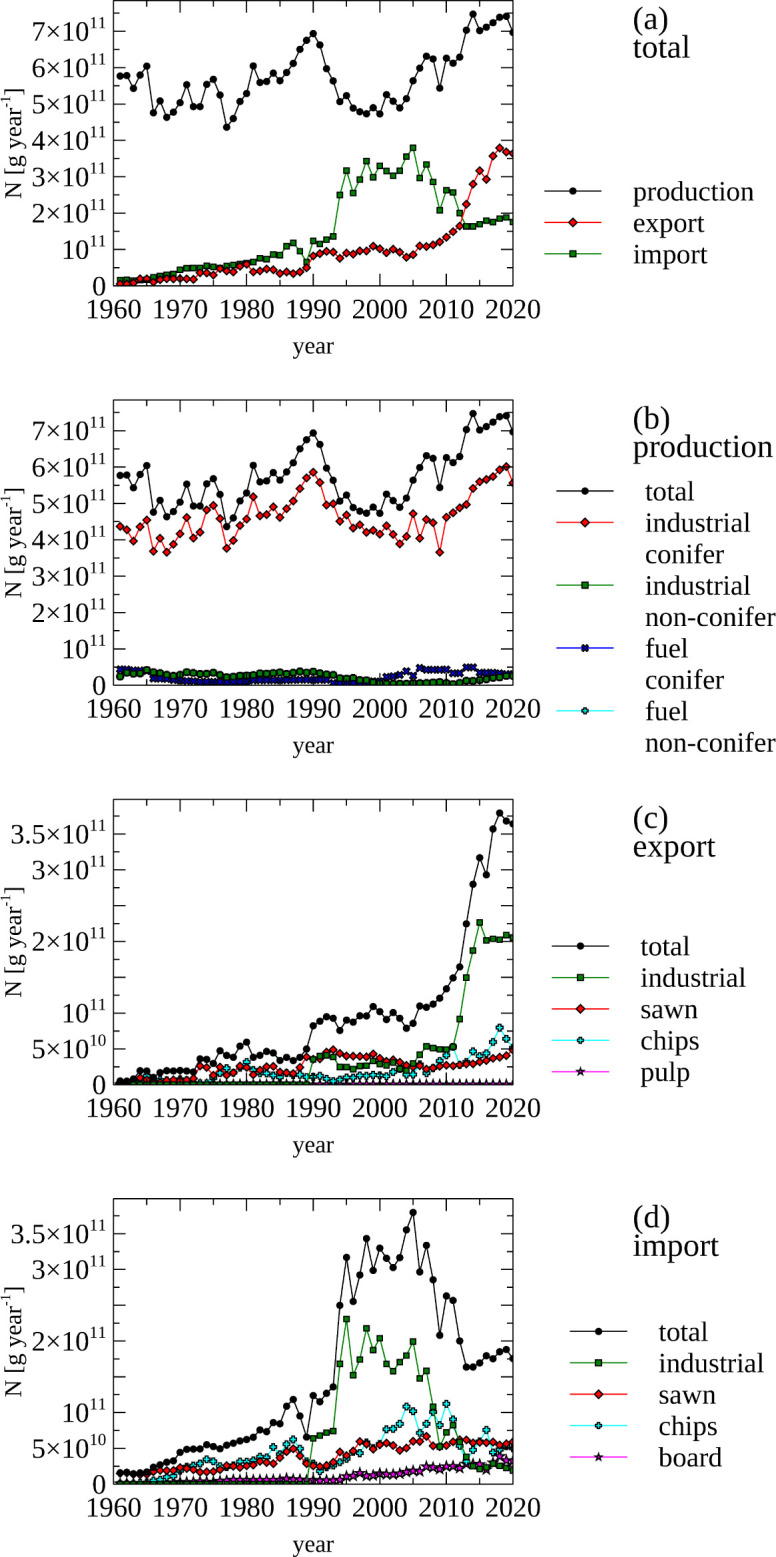
Forestry—amount of nitrogen in produced, imported and exported wood products, total and by product category. (a) Total amount of nitrogen contained in produced, imported and exported wood products. (c, d, e) Total amount of nitrogen contained in produced (a), exported (b) and imported (c) forestry products grouped into product categories. Note the different scales for panel a & b and panel c & d.

The total amount of N contained in the harvested wood is presented in Fig 3 panel a. There was an increase of N contained in imported wood (early 1990s to early 2010s), which declined concurrently with increased N production and N export. Coniferous industrial wood dominates N contained in harvested wood (Fig 3, panel b), while industrial non-coniferous wood and fuel wood are only minor sources of produced N.

Industrial wood and fuel wood are the only production items. However, a number of wood products are traded that also contain N. Sawn logs have the same N content as the logs they were derived from. Wood pulp, however, does not contain N, as the N is removed in the pulp production and this N must be industrially processed or contributes to the N load of sewage [51,88].

Since the 2010s export of N contained in wood has increase from containing ~100 Gg N year^-1^ to ~350 Gg N year^-1^ (Fig 3, panel c), with industrial logs, sawn logs and wood chip being the largest exported sources of N.

There was a short increase in imported industrial logs (early 1990 to early 2010). Currently, however, sawn logs, wood chips, and board products are the main sources of imported N in wood products (6. Forest panel d) containing ~180 Gg N during the late 2010s.

### Fisheries

The long coast and the proximity to very productive fishing grounds is the basis of Norway’s fishing history the strong fisheries sector today. As of 2018/2019 around 8500 people are full-time employed in the fisheries production sector [89], and employment in the seafood sector, including processing accounts for a total of ~31400 full time jobs [90].

Fisheries products can be categorized into two groups, wildly caught products and products that have been maintained in aqua-cultural settings. Traditionally, Norwegians used boats and ships to capture fish in fjords, along the coast, and on the high seas. However, in the mid 1990 aquaculture of sea water-tolerant fish, in particular salmon (especially the Norwegian Atlantic salmo, *Salmo salar*) and trout (especially rainbow trout, *Oncorhynchus mykiss*), started.

**Fig 4:**
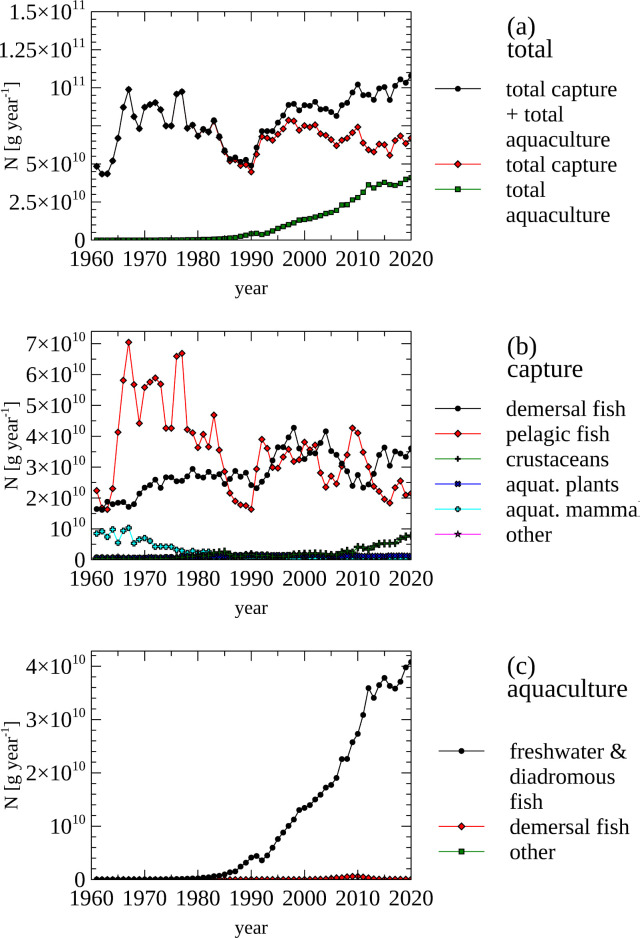
Fisheries—Nitrogen content of fisheries products. (a) The total amount of nitrogen contained in fisheries products from capture and total aquaculture. (b) Nitrogen content of products from capture by product category. (c) Nitrogen content of products from aquaculture by product category.

FAO Fisheries data between 1961 and 2020 provides insights in the amount of N that is contained in fisheries products, which include fish, crustaceans, aquatic mammals, mollusks (including cephalopods), echinoderms, as well as aquatic plants (Fig 4).

In recent times, the total amount of N produced in fisheries (107 Gg N year^-1^) is mainly based on fish, while in the early 1960s aquatic mammals contributed (~ 9 Gg N year^-1^) about 1/5 of the produced N (~45 Gg N year^-1^) (Fig 4, panel b). Sizable N output from aquaculture started in the mid 1980s, and has increase to 38 Gg N year^-1^ in 2018 (Fig 4, panel c). In addition to caught and farmed fish, the capture of other groups of animals and plants also contributes to the N output of fisheries. Here, the dominant N output is from crustaceans (e. g. shrimp) with 8 Gg N year^-1^, while aquatic plants, mollusks, cephalopods and echinoderms contribute less than 1Gg N year^-1^ combined (Fig 4, panel b).

The trading and domestic supply of fisheries products is detailed in a later section (Food Agricultural Products)

### Aquaculture and aquaculture feed

While fish caught in the open sea was Norway’s main source of fish in the 1960, fish grown in aquaculture and here in particular salmon and trout species are now approaching the amount of fish caught in the open sea. Aquaculture started this boom in the 1980, and concomitant with interest in feeding fish in aqua-cultural production, made Norway the leading producer of commercial fish feed.

The amount of protein used to feed salmon and rainbow trout in aquaculture in 2020 was 124 Gg N. As in 2020 the total amount of N in aqua cultural fish was calculated to be 40.9 Gg N, the protein retention rate can be calculated to 33%. As the retention rate is a species-specific parameter, and the feed is composed to match the physiology of the species, the supply of N in feed is proportional to the N contained in fish raised in aquaculture. This retention rate is in line with the 34% retention rate reported for salmon and rainbow trout [54,55]. Therefore, about 2/3 of the N supplied as food in aquaculture is lost into the coastal waters.

The trade of aquaculture feed is not included in FAO food and feed statistics. Norway is a main producers of aquaculture feed, even though the great majority of ingredients of this food are not produced in Norway and must be imported, as can be gleaned from the raw ingredients going into feed. Salmon feed is consists of soy protein concentrate (21.3%), sunflower expeller (6%), Wheat gluten (5.8%), Rapeseed (18.3%) oil, wheat (9.9%)), Fishmeal (19.5%) and fish oil (11.2%) [91].

### Non-food agricultural products

Non-food agricultural products are predominantly used for animal feed (livestock) but also include natural fibers, such as jute, wool and also rubber. Animal feed is mostly traded in the form macerated plant seeds, summarized as “seed cake”. These seed cakes are predominantly composed of soybean and rapeseed and imported into Norway.

**Fig 5:**
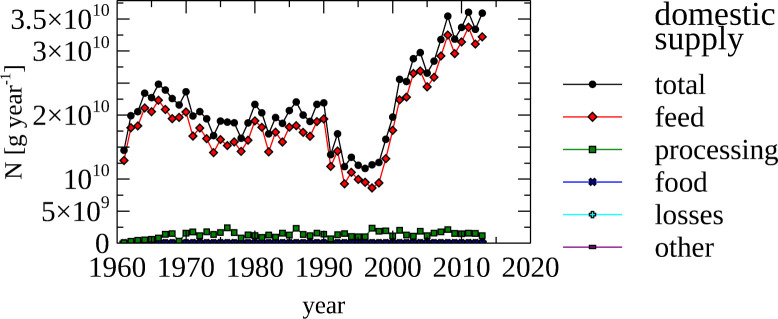
Non-food–Nitrogen contained in the domestic supply of non-food products. The amount of nitrogen contained in domestic supply of Non-Food products categorized by use between 1961 and 2020.

FAO data detailing production, export and import of non-agricultural products was parsed into groups of products, with “seed cake” and “fiber” the dominating products that were produced and traded. Product-specific N content were used to calculate the amount of N contained in these products. The domestic supply of N contained in non-food agricultural products (Fig 5), reveals that most N is used–as expected—for animal feed.

**Fig 6:**
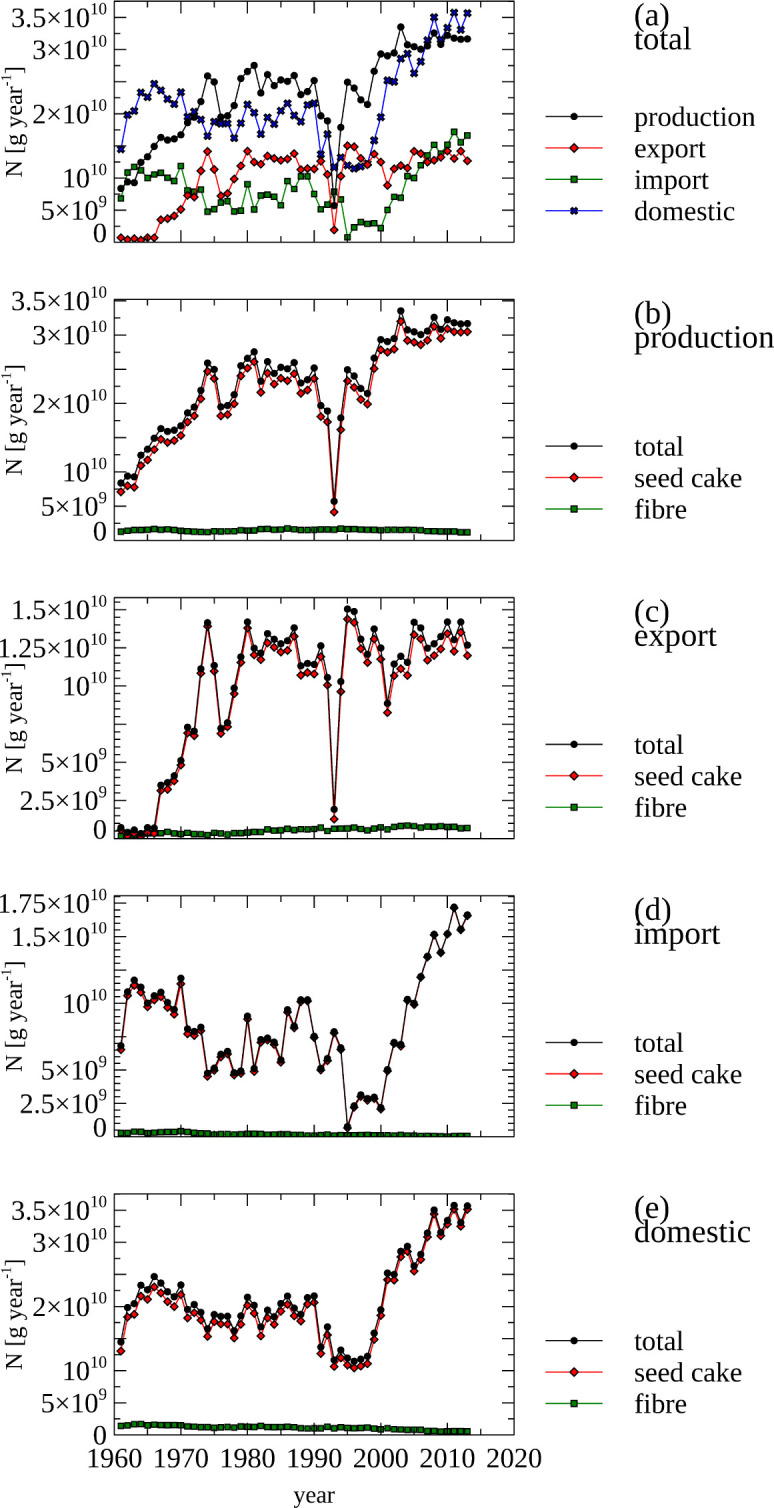
Non-food–Nitrogen contained in produced, imported, exported and domestically supplied non-food products, total and by category. (a) Total amount if nitrogen contained in produced, exported, imported and domestically supplied non-food products. (b, c, d, e) Total amount and categories amount of nitrogen in produced (b), exported (c), imported (d) and domestically supplied non-food products. Note the different scales used in panels a, b & e and panels c & d.

The total amount of N contained in non-food agricultural produced, traded and supplied for domestic use is detailed in Fig 6, panel a. The production of seed cakes is the main contributor to N contained in produced non-food products, increasing from 7 Gg N in 1961 to 32.5 Gg N in 2020 (Fig 6, panel b). In the same time period,the amount of N in fibre production remained constant at around 1.5 Gg year^-1^.

Export of N contained in seed cake increased from almost non-existent in 1965 to 12.5 Gg N in 2020 (Fig 6, panel c). The export of N contained in fibre increased from 135 Mg N year^-1^ to around 700 Mg N year^-1^ between 2010 and 2020.

Trends in N import of non-food products are dominated by seed cakes and are reflected in the domestic seed cake consumption, reaching a maximum in the year 2014 of 34 Gg N.

Another source of livestock feed is from items that are traditionally considered food are discussed in the Food section.

### Pasture feed and fodder

Pasture feed and fodder from agriculture includes items such as fodder (from hay and fresh grazing), as well as silage and some feed additions, such as gluten. In Norway, hay (mostly from Timothy grass) is harvested in late summer and provides a large store of feed for farm animals throughout the colder parts of the year. Grazing on fresh plants such as rye-grass, forage rape, and marrow-stem kale during the growth season also contributes to animal nutrition.

Two datasets are the bases for analyzing the N content in this animal feed (livestock). One dataset [58] reports domestic production, while the second [61] contains exported and imported amounts of feed items. The reported weights of each item were used to calculate the N content, using animal feed-specific conversion factors (see Table 2).

Norway produces most of the animal feed in this category for domestic use, with only small quantities imported and exported, which is reflected in the produced and traded amounts of N (S4 Fig, panel a). The amount of N in animal feed (livestock) that is produced in Norway is to a large extent contained in hay (S4 Fig, panel b), with around 40–45 Gg N year^-1^ used between 2000 and 2020. Silage and fresh grazing make up only a relative small amount of the total N consumed by farm animals.

Imported N is mainly contained in gluten, which is imported at a rate between 1 and 4 Gg N year^-1^. Interestingly, in recent years, a gluten was exported (0.700 Gg N year^-1^). Compared to gluten, there is is only a small amount of N traded in hay and feed vegetables.

### Live animal exports and imports

Even though FAO data for trade of life animals is available from 1961 onward, there are gaps in reporting within the earlier years of the data set, which make an analysis of this data over time unsuitable. However, data sets in more recent years are more complete and their average (for the years 2017–2019) for traded live animals and the contained N was calculated and to obtain an approximation for the year 2018. The main animals exported where horses, turkeys and chicken. The main animals exported where horses and swine. The traded amount of animals in fresh weight (325 t import / 143 t exported) corresponding to 6.5 Mg N (import) and 2.8 Mg N (export) for 2018.

### Livestock manure

**Fig 7:**
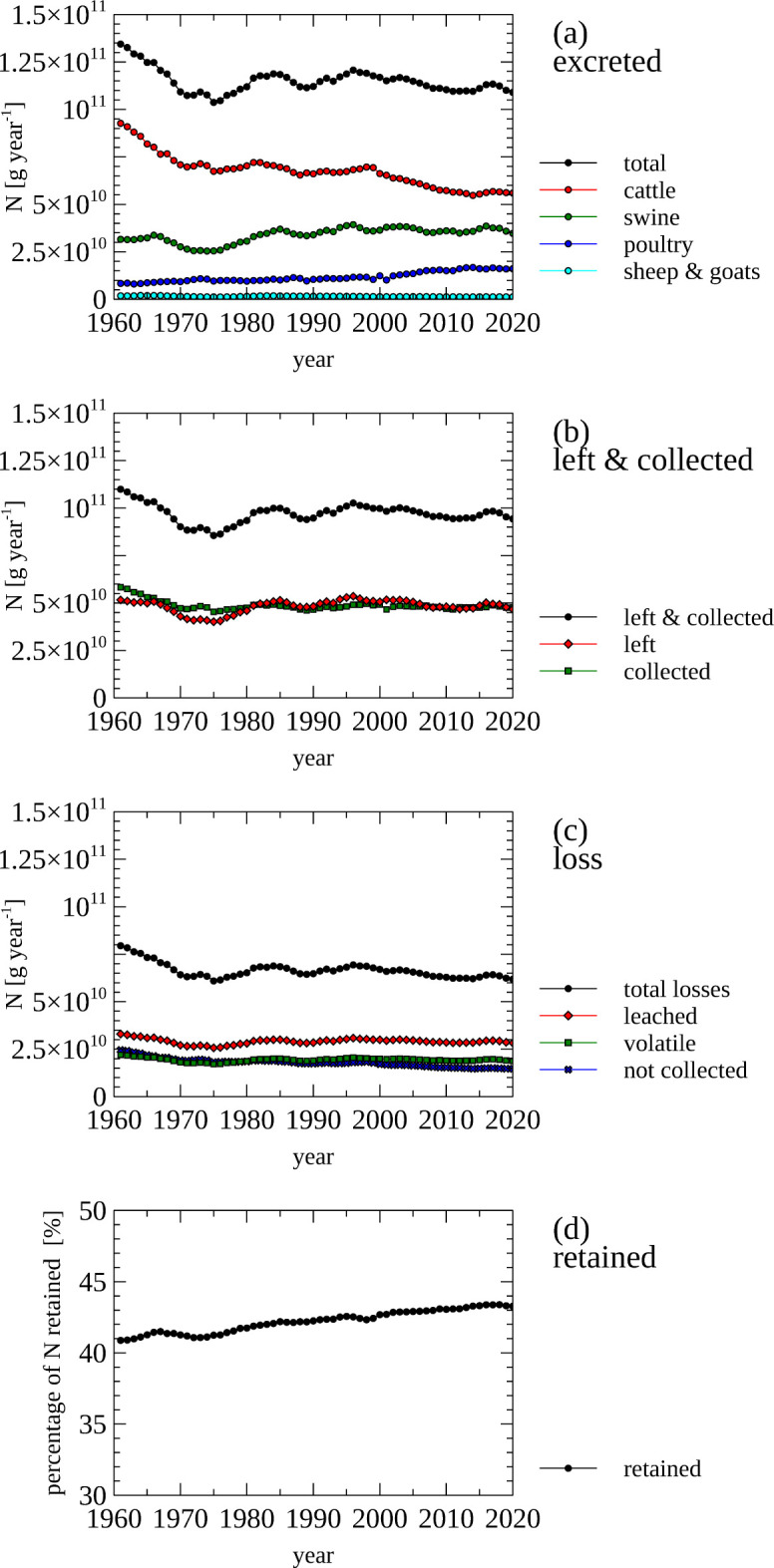
Livestock manure–Nitrogen inputs and losses from animal manure and retention. (a) Total amount of nitrogen excreted in the manure of all farm animals, and by animal species. (b) Total amount of nitrogen which is left on the pasture or collected/applied, and the left and collected/applied fraction (were added to calculate the total). (c) Nitrogen losses, including total nitrogen losses due to leaching, volatility and not collected and applied to soils. (d) Percentage of nitrogen that is retained (not leached, volatile and collected/applied to soils.

The manure-producing animals included in the data displayed in Fig 7 include dairy and non-dairy cattle, chickens layers and broilers, market and breeding swine, sheep, goats, horses, ducks, turkeys, buffalo camels, llamas, mules, as well as asses.

The total amount of N excreted by the surveyed livestock animals decreased between 1960 and 2020 from 135 Gg N year^-1^ to 110 Gg N year^-1^ (Fig 7, panel a)_._ This decrease is mainly due to a reduction of N excreted by cattle (dairy and non-dairy), while excretion form swine (breeding and market) remained stable, and N excretion increased for poultry (chicken broilers and layers, ducks and turkeys). The N excretion by sheep and goats and horses is only a minor contributor to total N excreted by farm animals.

The amount of N that is left on the pasture or collected for further processing (Fig 7, panel b) is lower than the total amount of N excreted (Fig 7, panel b). This non-collected fraction contributes to the total loss of N along with leached and volatile N (Fig 7, panel c). Leaching and volatilization occurs to manure that is left on pastures as well as manure that is collected, processed and applied to soils including cropped soils. The fraction of N that is retained (not lost) increased from 41% (1960s) to 43% (2010s) (Fig 7, panel d), indicating only a small improvement in agricultural practices for capturing N.

### Cropland nutrient budget–Nitrogen inputs and losses

**Fig 8:**
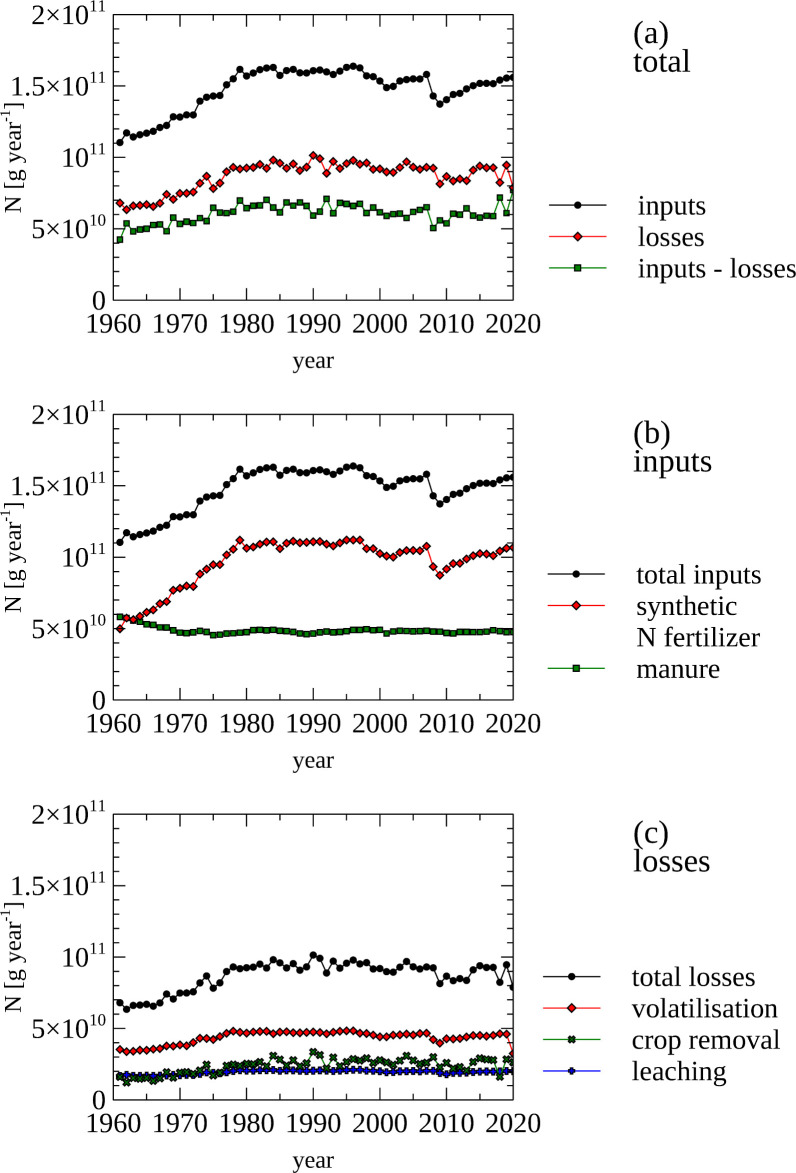
Cropland nutrient budget—Nitrogen inputs and losses on cropland land. (a) Total amount of nitrogen inputs, losses and difference between the inputs and outputs (b) Inputs of nitrogen, including the computed total nitrogen inputs from synthetic N fertilizer, application of manure, atmospheric deposition and biological nitrogen fixation (c) Losses of nitrogen, including the computed total nitrogen losses from volatilization, crop removal and leaching.

The data presented in Fig 8 represents the input of N from synthetic N fertilizer and manure onto cropland [32]. The total input of N onto cropped land from these sources increased from 110 Gg N year^-1^ (1960s) to 160 Gg N year^-1^(2020) (Fig 8, panel a). The total fraction of N retained from theses inputs during this time period (inputs / (inputs–losses) is around 0.42 (Fig 8, panel b). In the 1960s N input from manure and synthetic fertilizer contributed equally to total N input. While the N amount from manure remained stable, the input from synthetic fertilizer more then doubled by 1980 and remains at this level to the present day.

The FAO data set also contained data for atmospheric deposition (1.4 Mg year^-1^) and biological N fixation (0.25 Mg N in 2020). This data is at odds with the biome-derived estimates for N input from biological N fixation (79.9 Gg N year^-1^) and atmospheric deposition (50.7 Gg N year^-1^), even taking into account that the cropped area is only between 2–3% of the total land area of Norway. Therefore, these data sets (biological N fixation and atmospheric N deposition) were not displayed in the graphs and are not used in further analysis.

Of the sources of N loss, through crop removal, leaching and volatilization (Fig 8, panel c), leaching could be most decreased over the investigated time period. A detailed analysis of organic and inorganic N inputs representative for 96% of the N used for crops for Norway was recently constructed [92].

### Food agricultural products

The FAO [59] data includes items that are traditionally used for food. However, some of these items do not necessarily end up on a plate, but in a trough as animal feed, or may be further processed or lost.

**Fig 9:**
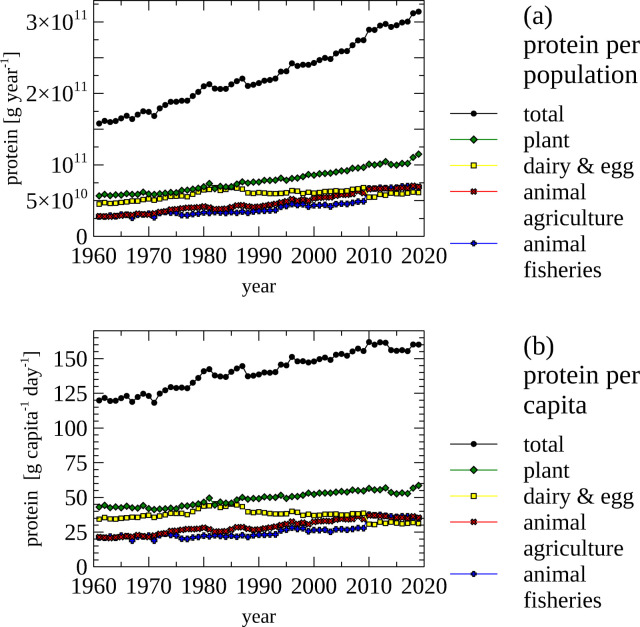
Food—protein supply for the population and per capita, total and by food group. (a) Protein contained in food supply for the population of Norway, total and by food group. (b) Protein contained in food supply per capita, total and by food group. Note the different scales used in panel a and panel b.

The amount of N contained in food is generally reported as the amount of protein. Following this convention the amount of protein provided by the domestic food supply is shown (Fig 9). However, these data do not indicate consumed protein, but the protein in products supplied for food, which are of course related. The amount of protein available in the food supply for the population of Norway (Fig 9, panel a), as well as on a per capita bases (Fig 9, panel b) was calculated, and mapped to different food groups.

The amount of protein in the food supply per person increase from ~120g day^-1^ in the 1960s to ~ 160 g day^-1^ in the 2010s. Again, it is important to distinguish between the protein that is supplied as food and the actual food intake. The protein intake is reported to be 116.2 g protein person^-1^ day^-1^ [59] in 2018, while the protein contained in supplied food for that year was calculated to 160.2 g protein person^-1^ day^-1^. The difference is due to losses, such as food waste. The protein consumed in plants and animals produced in fisheries increased steadily between 1961 and 2020, while consumption of milk and eggs decreased since the mid 1980s. The consumption of protein from animals originating from agriculture has plateaued since 2010.

**Fig 10:**
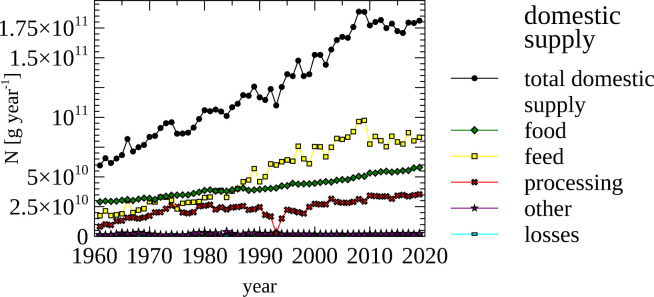
Food—Nitrogen contained in domestic supply of food products. The total amount of nitrogen in all food products was calculated from all use categories, which are also shown.

A look at the domestic supply of food products on an N-basis may surprise, as more than half of items in the FAO food category is presently used as animal feed (Fig 10) (80.25 gG N year^-1^). The cross-over-point—when more items in the FAO data set were used as feed than food—was in the mid 1980s. This change can be attributed to a shift in dietary preferences and an increase in population. The traditionally consumed cattle and sheep can live off grass and hay, while swine and poultry, which are proportionally consumed more in recent times, require a different feed. Here, grains and grain-derived products are used for poultry, while swine also consume root vegetables, which are can also be consumed by humans.

**Fig 11:**
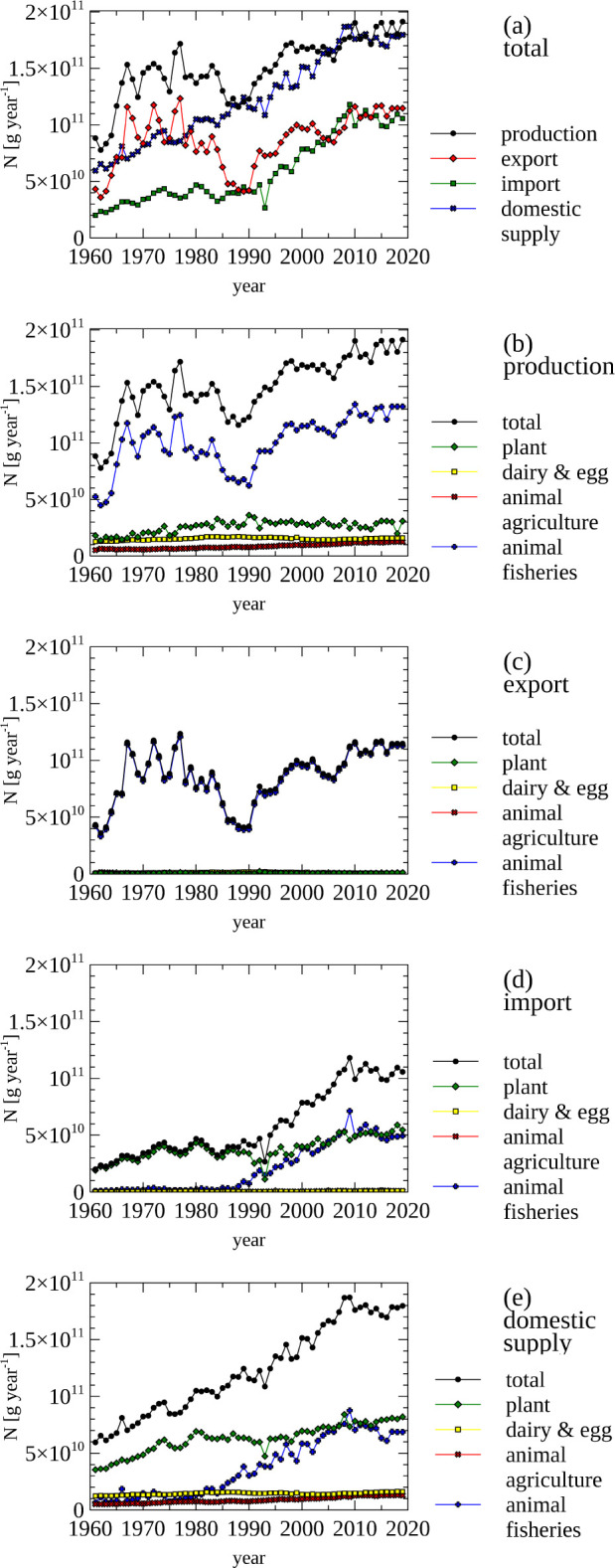
Food—Nitrogen contained in imported, exported, produced, domestically supplied food products by food group. (a) Total nitrogen contained in produced, exported, imported food products, and nitrogen contained in food products in domestic supply. (b, c, d, e) Total amount of nitrogen contained in produced (b), exported (c), imported (d), and domestically supplied (e) food products, as well as the individual components grouped by origin.

The total amount of N contained in food that is produced and traded and domestically supplied is shown in Fig 11, panel a. Between 1961 and 2020, the amount of imported N and domestically supplied N increased, stabilizing at around 125 Gg N (import) and 180 Gg N (domestic supply) in the 2010s. While more N contained in food items was exported between the mid 1960s to the mid 1980s, when supplied domestically, export and domestic supply match each other. The N that supports this export of food items is not based on domestically produced N, but on off-shore resources in the form of captured fish, and in N contained in (imported) fish feed. Overall it is remarkable that Norway, as a net importer of N in food/feed, can export large amounts of N through the fisheries sector based on wild capture and import of aquaculture feed.

The dominance of fisheries in the production of N in food can be gleaned from graphs that show production data (Fig 11, panel b) and export data (Fig 11, panel c), which dis-aggregates the total of produced N into food categories. Food exports from fisheries (~115 Gg N year^-1^) dwarf exports from the agricultural sector (~1 Gg N year^-1^) in the late 2010s).

N contained in items in the food category are imported in the form of plants (~50 Gg) and fishery products (~54), while very little milk, eggs and farm animal products are imported (in the late 2010s).

It may be expected that proportion of food categories in the domestic supply data (Fig 11, panel e) matches the food categories in per capita protein consumption (Fig 10). However, when comparing the corresponding graphs is noticeable that much more fisheries products are supplied than are consumed by humans. This is not an effect of the used units ([g protein day^-1^] vs [g N per year]), as these are related by a conversion factor, but by the fact that items in the FAO data are also used as animal feed.

### People

To put amounts reported in Giga grams (Gg) in perspective, looking at the flow of N in people provides some perspective. The amount of N contained in a person is ~1.5 kg. Net migration of ~18000 people in 2018 results in an N input of a mere 27 Mg in 2018.

### People–Nitrogen emission through skin and by breathing

Since farm animals are a main emitter of ammonia, a look at emissions from another dominant mammal in Norway may be of interest. Much of human excreted N is processed by the sewage system (see next section), however the amount of N emitted through skin and by breathing is substantial. This emission amounts 333 Mg N year^-1^ for people living in Norway in 2018.

### Sewage

The wastewater, which contains the left-overs of human digestion, is a valuable source of N and other nutrients. In Norway most (87% in 2018) of the population is connected to a sewage system. Sewage is treated using a variety of methodologies, including chemical, biological and mechanical treatments and combination thereof [75].

Data for loss/discharge of N from all sewage plants (municipal sewage, as well as smaller plants) was taken from Statistics Norway [73], which covers the years 2003–2020 (S5 Fig). The loss of N increased from ~16 Gg N in (2003) to ~20 Gg N (2020). Taken the entire population of Norway, the loss of N in sewage per capita increased from 3.51 kg N year^-1^ (9.61 g N day^-1^) in 2003 to 3.69 kg N (10.11 g N day^-1^) in 2020. After sewage treatment, the N dissolved in sewage is discharged into streams and coastal waters, however, a proportion of the N in sewage can be recovered as sewage sludge.

### Sewage sludge

In the year 2018, the 111.7 Gg (111.7 kt) dry sewage sludge was recovered from sewage in Norway. Of this sewage sludge, 92.1 Gg was used to enrich soils, including pastures and agricultural areas. In the same year, 6.7 Gg of sewage sludge were used as filler for waste sites and 12 Gg have other uses. Only 1.1 Gg of sewage sludge was of a quality that required storage in a landfill. Assuming an N content of 2.5% for dry sewage [93], it is possible to calculate the approximate amount of N contained in sewage sluge. The total amount of N contained in sewage sludge is 2.8 Gg N with 2.3 Gg N being used to re-enrich soil and only 28 Mg N were considered of poor quality requiring special storage, in 2018. This indicates that the amount of N contained in sewage sludge is only 12% of the ~20 Gg of N that are lost/discharged into rivers and the ocean in 2018.

### Waste

Much of waste that has high energetic value, such as plastics and paper, has little N content and is burned to generate electricity in Norway. The N content of this waste is therefore reflected as NOx emissions in the “energy generation” category (see NOx and Ammonia Emission Section). The N-containing waste stream that is not burned consists mostly of bio- waste and contained about 28.3 Gg N in the year 2018, here food waste is estimated to make up about half the amount of N, the other half stemming from the categories “wetorganic waste”, “park and garden waste” and “wood waste”. This waste is considered to be used to enrich agricultural soils.

### Nitrogen oxide and ammonia emissions

Two classes of air-borne molecules constitute the main N-containing air pollutants and are therefore monitored extensively. Chemically oxidized N is bound to oxygen, forming nitrogen oxides (NOx), and chemically reduced nitrogen is bound to hydrogen, forming ammonia (NH_3_).

Molecules of NOx are produced when N-containing organic molecules are burned. Burning of fossil fuels, as well as burning of wood contributes the majority of NOx emissions into the atmosphere along with natural sources, such as forest fires and lightening.

The main source of ammonia is from livestock. Here, ammonia is produced from the protein metabolism and primarily excreted in feces and urine by mammals and birds. A much smaller amount of ammonia is exhaled and excreted through skin.

**Fig 12:**
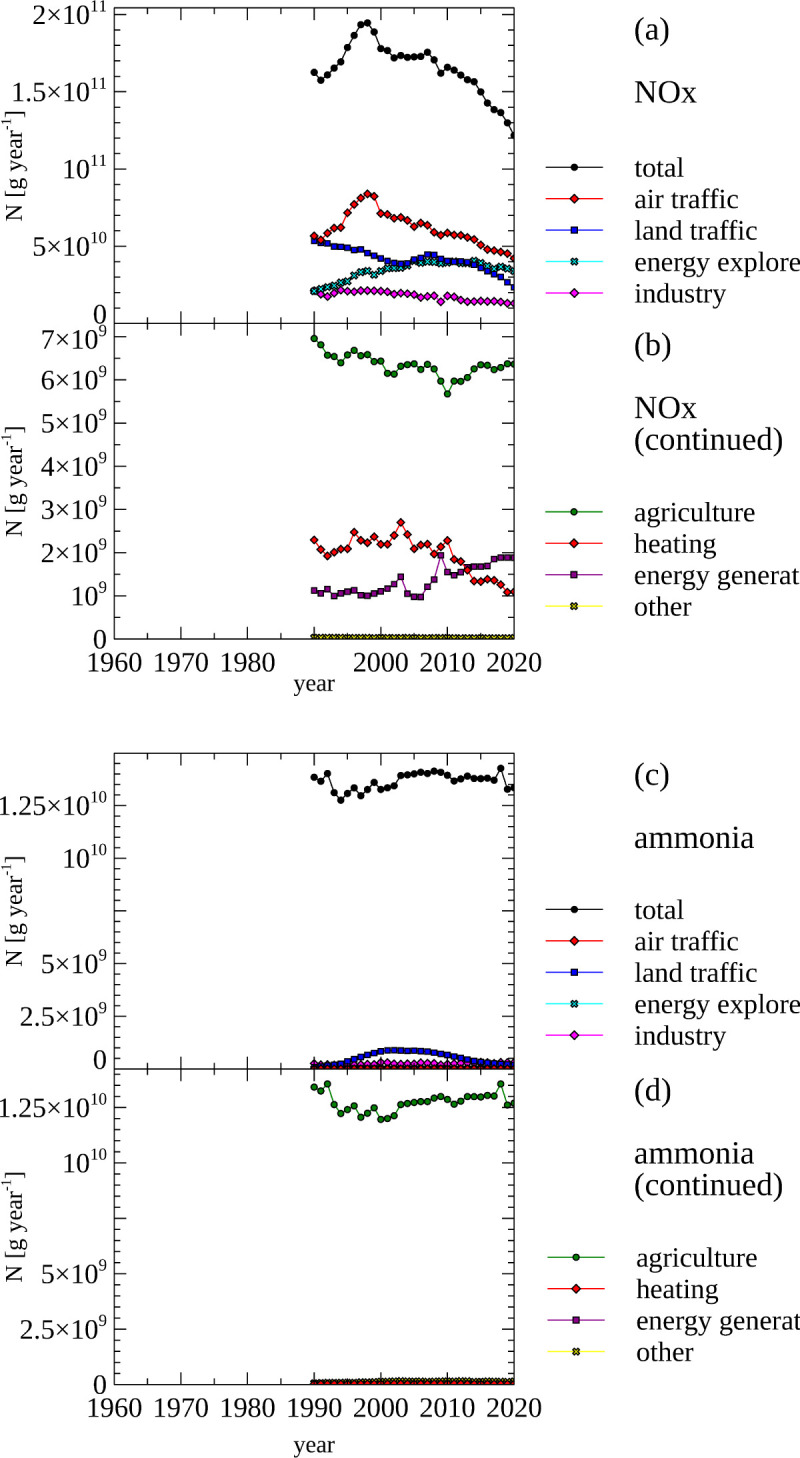
Nitrogen oxide and ammonia emissions–amount of nitrogen contained in volatile emissions, total and by sector. (a, b) Total and sector-specific amount of nitrogen emitted in NOx. (c, d) Total and sector-specific amount of nitrogen emitted in ammonia. Note the different scales used in panel a and panel b, as well as between panels c and panel d.

The source of N emissions in the from of NOx and ammonia are detailed in Fig 12 based on a Statistics Norway data set [79] spanning the years 1990 to 2020. This data set originally reported emissions using the weight of the emitted molecules. In the presented graph the weight of N contained in NOx and ammonia was calculated from the atomic composition of these molecules.

NOx is the main air-borne N-containing components emitted in the year 2020 in Norway with 12 Gg N, followed by 7.4 Gg N of ammonia.

Total N emissions from NOx were highest in 2005 (19 Gg N) and have decreased by around a third since (Fig 12, panel a & b). The main drivers for this trend are declined emissions from traffic (air and land), as well as industry. Another major source of N emissions is the exploration of fossil energy. Minor sources of N emissions (smaller than 2 Gg N year^-1^) are NOx emissions from agriculture, heating and energy generation.

The dominant source of ammonia-based N emissions is agriculture at around 13.5 Gg N year^-1^ (Fig 12, panel c & d). The sum of all other N emission from ammonia is less than 1.1 Gg N year^-1^, which is mostly through emissions from land traffic and industry.

## Material and products

This study dos not include a analysis of material and products other than then ones discussed above. This gap can be justified by examining S6 Fig that reports import and export in monetary terms. This figure reveals that most traded commodities that contain substantial amount of N are covered in the data analyzed in this study. A few items that contain N and are not included in the study are of high value per weight (e.g furniture, pharmaceutical products), so that the total amount of N traded is small. There is uncertainty in assessing the N footprint of metal and metal ore processing. Here, to account for N flows, the resources used for metal and metal ore generation, and how these resources are reused or discarded should be considered in the future.

### Flows of nitrogen in Norway in the year 2018

**Fig 13:**
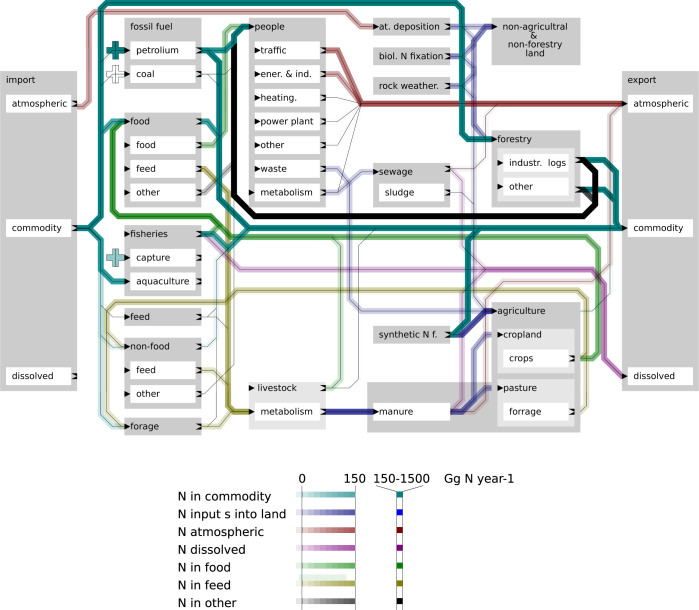
Flows of nitrogen in Norway in the year 2018.

The flow of N in Norway for the year 2018 is presented in Fig 13. This year has been chosen at it presents to most recent year where all data necessary to construct and cross-validate the overall flow of N was available. The N flows are discussed in the following section and also available in a separate file (S1 Data) that also contains all relevant calculations.

The flows are grouped by categories indicated by colors. All nitrogen flows are indicated with the same width and a centered thin black line, both of which are independent off the the amount of nitrogen flow. The amount of nitrogen flow is indicated by the color intensity (see legend). Consequently, small flows will only be discernible by a thin black lines. Small losses in flow are not indicated. The flow of chemically inert, atmospheric nitrogen (N_2_) is not indicated. The flow values can be found in the the text (section: Flows of nitrogen in Norway in the year 2018) and are also contained in a file (S1 Data). Abbreviation: synthetic N f.–synthetic nitrogen fertilizer; rock weather.—rock weathering. Please note that biological nitrogen fixation.

#### Nitrogen inputs

The N inputs into Norway are from atmospheric deposition (50.7 Gg N year^-1^), biological N fixation (79.9 Gg N year^-1^) and rock weathering (47.6 Gg N year^-1^), totaling 178.2 Gg N year^-1^. Most of these inputs are partitioned onto non-managed land areas (92.8 Gg N year^-1^), while managed forests and agricultural land received N inputs of 61.2 and 22.2 Gg N year^-1^, respectively.

The input of N into agricultural land through the use of synthetic fertilizer (102.4 Gg year^-1^) is higher than those by atmospheric deposition, biological fixation and rock weathering combined (22.2 Gg N year^-1^). Synthetic N fertilizer is the commodity with the largest amount of N (554.2 Gg) exported in 2018.

#### Fossil fuels

Domestically consumed petroleum contains 133.19 Gg N year^-1^_,_ while 229.54 Gg N year^-1^ are exported. A small net amount of N contained in coal is imported (12.66 Mg N year^-1^) and along with produced coal used domestically (15.67 Mg N year^-1^). Both coal and petroleum products release NOx when consumed in traffic, industry, heating, or power generation (see NOx and Ammonia Emissions).

#### Forestry

The predominant forestry product is industrial logs. Industrial logs are further process in sawn logs, and other products, including wood chips. In 2018, most of the produced logs were used within Norway (containing 437.49 Gg N), while 202.40 Gg N contained in logs were exported. Other forestry products containing 106.74 Gg N were used domestically and 176.84 Gg N was exported in the same year.

#### Fisheries

The total amount of N in domestic fisheries production is assessed to be between 132.5 [59] and 100.6 Gg N year^-1^ [52, 5359]. Using the FAO fisheries data 63.4 Gg N year^-1^ comes from offshore capture and 37.19 Gg N year^-1^ from aquaculture. In order to grow the aqua-cultural products, feed containing 124.0 Gg N year^-1^ is imported. Thus only about 1/3 of the feed is retained in products, while 2/3 of N is released into streams and coastal waters. Of the total 132.5 Gg N year^-1^ in fisheries products [59] produced and ~48.8 N Gg N year^-1^ imported, ~68.6 Gg N year^-1^ are used in the domestic food supply, while a total of 112.7 Gg N year^-1^ are exported. It is assumed that it is in most cases not economical to export fisheries products that were imported, and thus the entire export is assigned to come from domestic capture and aquaculture.

#### Agriculture–animals

The main flows of N in agriculture comes from the feed required to sustain livestock. The main category for feed is from items that are traditionally considered food items (Food) (80.25 Gg N year^-1^). Here, the dominant source of N is contained in fish (1477 kt), followed by grain products (713 kt), aquatic plants (167 kt) and of pulses (133 kt) and potatoes (24 kt). A second source of feed is imported (feed), which consists mainly of seed cake and gluten (4.17 Gg N year^-1^). A third source of feed is contained in the “Non-food” category, which includes imported commodities (16.62 Gg N year^-1^) and feed items grown on domestic cropland (31.67 Gg N year^-1^), totaling 48.29 Gg N year^-1^. A fourth source of feed, which can be utilized by ruminant animals, is domestically produced forage such as hay and grass, containing 31.46 Gg N year^-1^. In aggregate, the total input into agricultural animals is 164.17 Gg N year^-1^, where only 28.54 Gg N are retained in animals and animal products used as food_._ The N conversion rate for livestock products can thus be calculated to be 0.174 (17.4%). This conversion rate is in line with a first principle calculation based on protein retention rates (see S1 Data–livestock tab).

#### Agriculture–manure

Another N resource generated by livestock is manure, which is estimated to contain ~112.35 Gg N in 2018. The difference between accounting of N input (feed: 164 Gg N year^-1^) and N output (animal products: 28.50 Gg N year^-1^, and manure: 112.35 Gg N year^-1^) is not due to export (0.314 Gg N year^-1^ of animal products) or trade in life animals (0.004 Gg N year^-1^). Manure produced by livestock is left on pastures (49.27 gG N year^-1^) or collected and applied to cropland (48.22 Gg N year^-1^). However, about half of N that is left and applied to agricultural land leaches into streams (29.25 Gg N year^-1^) or is volatilized as ammonia (19.50 Gg N year^-1^).

#### People–food

The Norwegian population has access to 57.55 Gg N year^-1^ contained in food—mostly in the form of protein. However, only 32.1 Gg N year^-1^ are consumed with most of the rest becoming N contained in food waste. Of the consumed N, only 0.33 Gg N are lost through skin and breathing, and most of the N is processed through sewage systems. In Norway 87% of people were connected to a sewage system, which thus processes ~27.94 Gg N year^-1^. Of this sewage 2.8 gG N year^-1^ is recovered as sewage sludge, while 19.4 Gg N year^-1^ (dissolved) and 6.21 Gg N year^-1^ (volatile) are not recovered. Most of the N contained in the sewage sludge (2.3 Gg year^-1^) is used to enrich agricultural lands.

#### People–waste

Waste suitable to enrich agricultural soils contained ~28.3 Gg N in 2018. The origin of this biowaste stems from the food supply, as well as garden and wood waste.

#### People—Nitrogen oxide and ammonia emissions

Human-made atmospheric N emissions are mainly from the consumption of fossil fuels, which predominantly produce NOx. The main source of fossil fuel-based N emissions is from transportation (76.64 Gg N year^-1^), followed by activities related to the exploration of energy and industrial activities (50.97 Gg N year^-1^), energy generation (1.89 gG N year^-1^) and heating (1.29 gG N year-1), and a small contribution by other N emission sources (0.148 gG N year^-1^). Atmospheric emissions by agriculture were detailed above.

#### Import and export

The total amount of N exported from Norway in 2018 as commodities was 1417.84 Gg N, while 504.77 Gg N was imported.

## Conclusions

The element nitrogen can serve as tracer for the flow of matter through the world. The relatively high abundance of N in biological material make N an especially suitable and reliable reporter of flows of this group of materials. For example, it is possible to connect the amount of petroleum consumed with the amount of NOx that is emitted. Furthermore, it is also possible to calculate the amount of protein retained in animal products from the amount of protein consumed in feed and excreted. As N cannot be converted into other elements all flows must balance and if they do not, this reveal gaps in our models and data. The presented study shows and discusses N flow in Norway from 1961 to 2020. For the year 2018 64 individual N flows have been resolved.

Does this study constitute an N budget for Norway? The goal of this study was to investigate the flows of N in a historic context and to allow to “go back in time”. Thus, it must be straight-forward to assess how a particular N flow has changed over time. To accommodate this, the data is discussed and presented as separate, origin specific and use-specific data sets. For example, all the sources of animal feed have not been consolidated, but are treated in reference of their respective data source (e. g. “Food”, “Non-Food”, “Feed”, and”Forage”). While this dis-aggregation allows flexibility in order to satisfy academic curiosity, in national nitrogen budget, one may wish to have these sources consolidated.

A properly designed N budget for a country should follow the UN guidelines for such a budget [11]. From the dimensions (“pools”) covered by these guidelines, the study presented here provides insights into the pools “Energy and Fuels”, “Humans and settlements”. The pool “Agriculture, Forest and semi-natural vegetation including soils”, is well resolved, with the exception of soils, where complete data on N cycling and N stocks in soils were not available. Furthermore, other pools are only covered cursorily in this study. For example, “Waste” is a complex topic that is also changing rapidly. What once was considered waste is becoming a resource in an increasingly resource-constrained world. This will surely mean that better data on the flow if N in waste streams will become available. While in the “atmosphere and hydrosphere” pool, atmospheric data is available and should allow to generate a model that is superior to the biome-dependent model employed in this study, data on the hydrosphere is harder to gather and therefore sparse. For the “Material and Products” dimension, this study only selected the major N-containing commodities (fossil fuels, food and feed, fisheries and forestry products, and synthetic N fertilizer). This choice is based on available data sets reporting gravimetric units, while commodities are often reported in monetary values, requiring conversions between these units that carry a high degree of uncertainty.

With the aforementioned limitations in mind, the present study nonetheless addresses the two motivations for national N budgets mentioned in the introduction. This study assesses and visualizes N flows, and thus provides a useful basis for policymakers and national experts to formulate policies that direct the use of N-containing resources.

*The more fodder, the more flesh’; the more flesh, the more manure*;

*the more manure*, *the more grain*!*”*

attributed to one of the leaders of English agriculture

by von Liebig, 1840 [87]

While von Liebig, the “father of the fertilizer industry”admired Japanese peasants for their agricultural practices, he could only smile at the ‘wisdom’ of self-declared experts at the time (cited above). N is a precious resource that is limited and its careful conservation, and limitation of N losses is crucial for maintaining a productive agriculture. “More, and more, and more” is a delusional view of agriculture that may have been smiled upon in the past, but should prompt anger at present. We live in a world, where the biomass of our livestock exceeds the biomass of all wild mammals by a factor greater than 10 [94], where the mass of human-made materials equals the entire biomass [95], and where consequently a mass extinction is well on its way [96]. The element N is a tracer of the human impact on our planet, and tracing the flow of N can help us to understand, how we must limit our impact in the future.

## Supporting information

S1 Table Python scripts and their functionality. (ODS)

S2 Table Nitrogen content of food, feed and other items. (CSV)

S1 Scripts Source code of Python scripts used for calculating N flows. (ZIP)

S1 Data N flows within Norway for the year 2018. This files details calculations that provide data displayed in Fig 13.
(ODS)

S1 Fig Agriculture land use and forest cover in Norway. The areas are shown in % of total land area of Norway as well as in km2. All available data is shown with most data (forest total, forest productive, cropland + pasture, cropland + pasture in use, pasture) not covering the entire time period (1961–2021).
(EPS)

S2 Fig Biome map of Norway. Biomes are assigned to areas of Norway using data from Olsen et al. 2001 [31].
(TIFF)

S3 Fig Forestry—volume of tree logs felled. The data for the volumes of logs from felled trees from two different data sources (FAO [46] and SSB [47]).
(EPS)

S4 Fig Pasture feed and fodder–pasture feed and fodder produced and traded, total and by category. (a) Total amount of N contained in pasture feed and fodder, produced and traded. (b,c,d) Total amount of N contained in produced (a), imported (b) and exported pasture feed and fodder by category.
(EPS)

S5 Fig Sewage—amount of N lost/discarded by sewage plants in Norway (2004–2020). The data represent municipal and smaller plants and also accounts for losses in piping.
(EPS)

S6 Fig Imported (top) and exported (bottom) commodities for Norway in the year 2018. The value of commodities is indicated by monetary value in USD (white bar at the top of the import and export section of the figure). The percentage of the value of each commodities is indicated by areas, assigned into colored categories (key at the bottom of the import and export section of the figure). Figures were constructed and downloaded from https://oec.world/en/visualize/tree_map.
(TIFF)
