# Supplementary figures and images for: A Nitrogen budget for Norway analysis of Nitrogen flows from societal and natural sources (1961–2020)
Source: PLoS One. 2025 Feb 13;20(2):e0313598. doi: 10.1371/journal.pone.0313598 (PMC11824958; doi:10.1371/journal.pone.0313598)

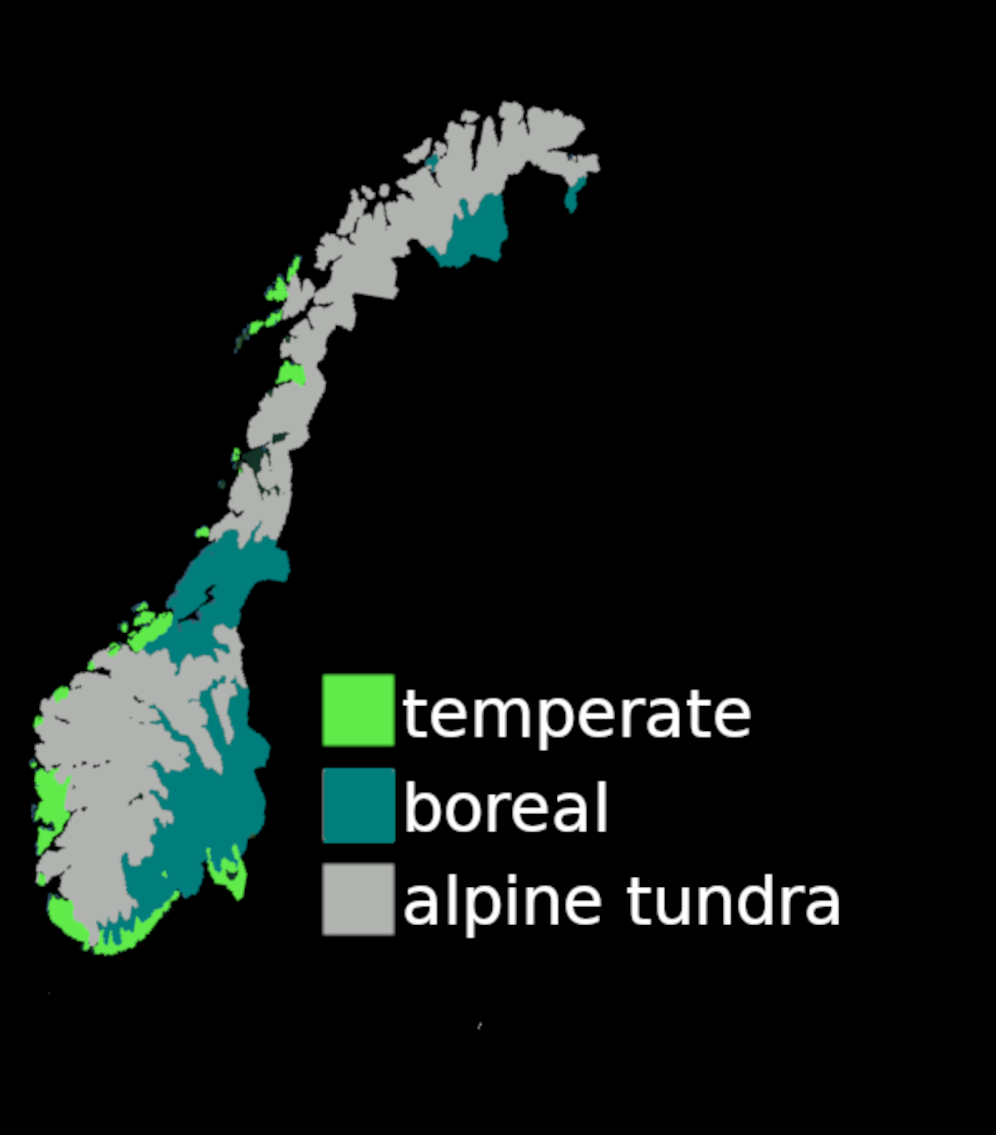

Supplement: S2 Fig — Biomes are assigned to areas of Norway using data from Olsen et al. 2001 [31]. (TIFF) [file pone.0313598.s006.tiff]

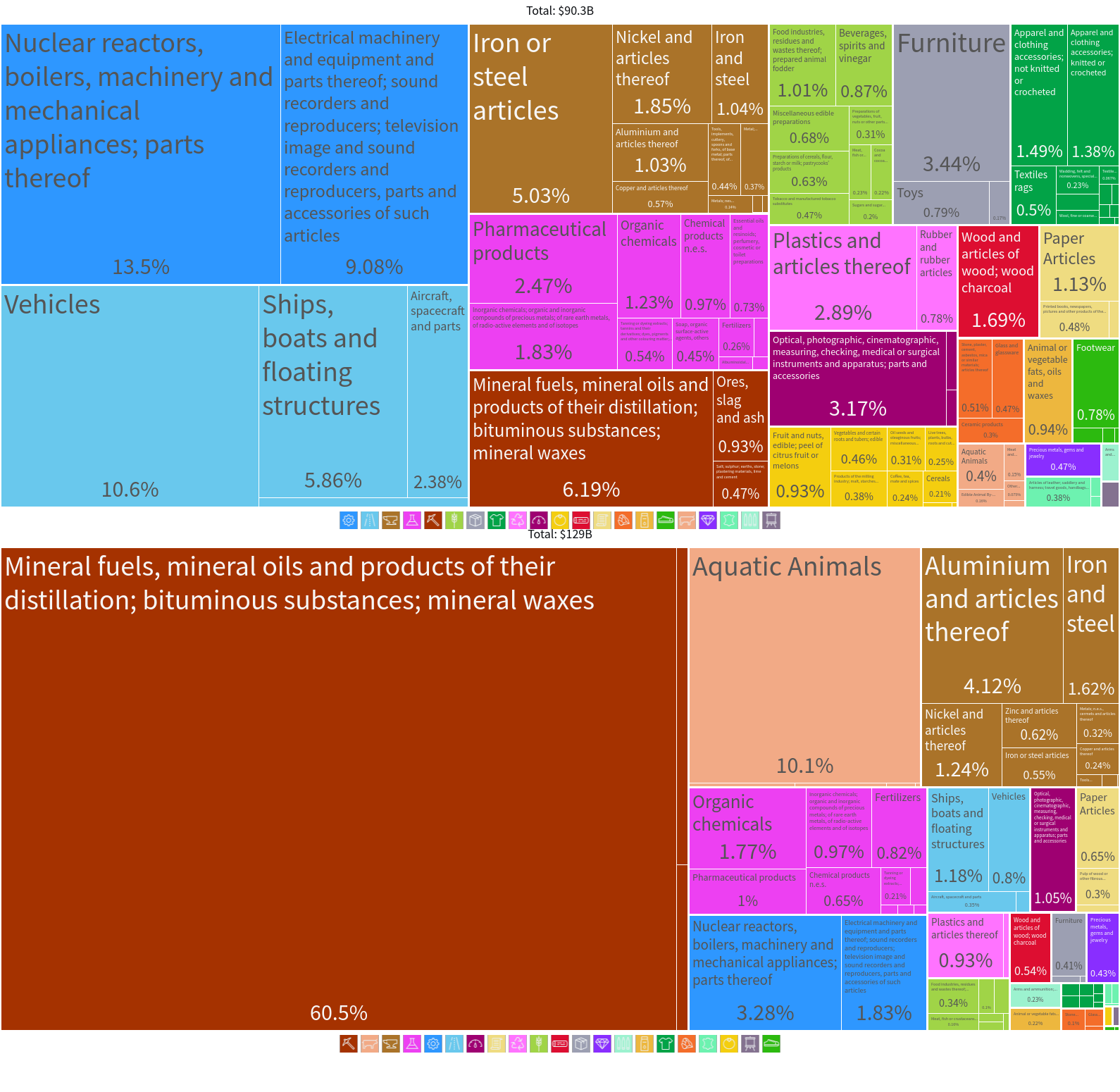

Supplement: S6 Fig — Imported (top) and exported (bottom) commodities for Norway in the year 2018. The value of commodities is indicated by monetary value in USD (white bar at the top of the import and export section of the figure). The percentage of the value of each commodities is indicated by areas, assigned into colored categories (key at the bottom of the import and export section of the figure). Figures were constructed and downloaded from https://oec.world/en/visualize/tree_map. (TIFF) [file pone.0313598.s010.tiff]
